# Supplementary figures and images for: Giant ankyrin-B mediates transduction of axon guidance and collateral branch pruning factor sema 3A
Source: eLife. 2021 Nov 23;10:e69815. doi: 10.7554/eLife.69815 (PMC8610419; doi:10.7554/eLife.69815)

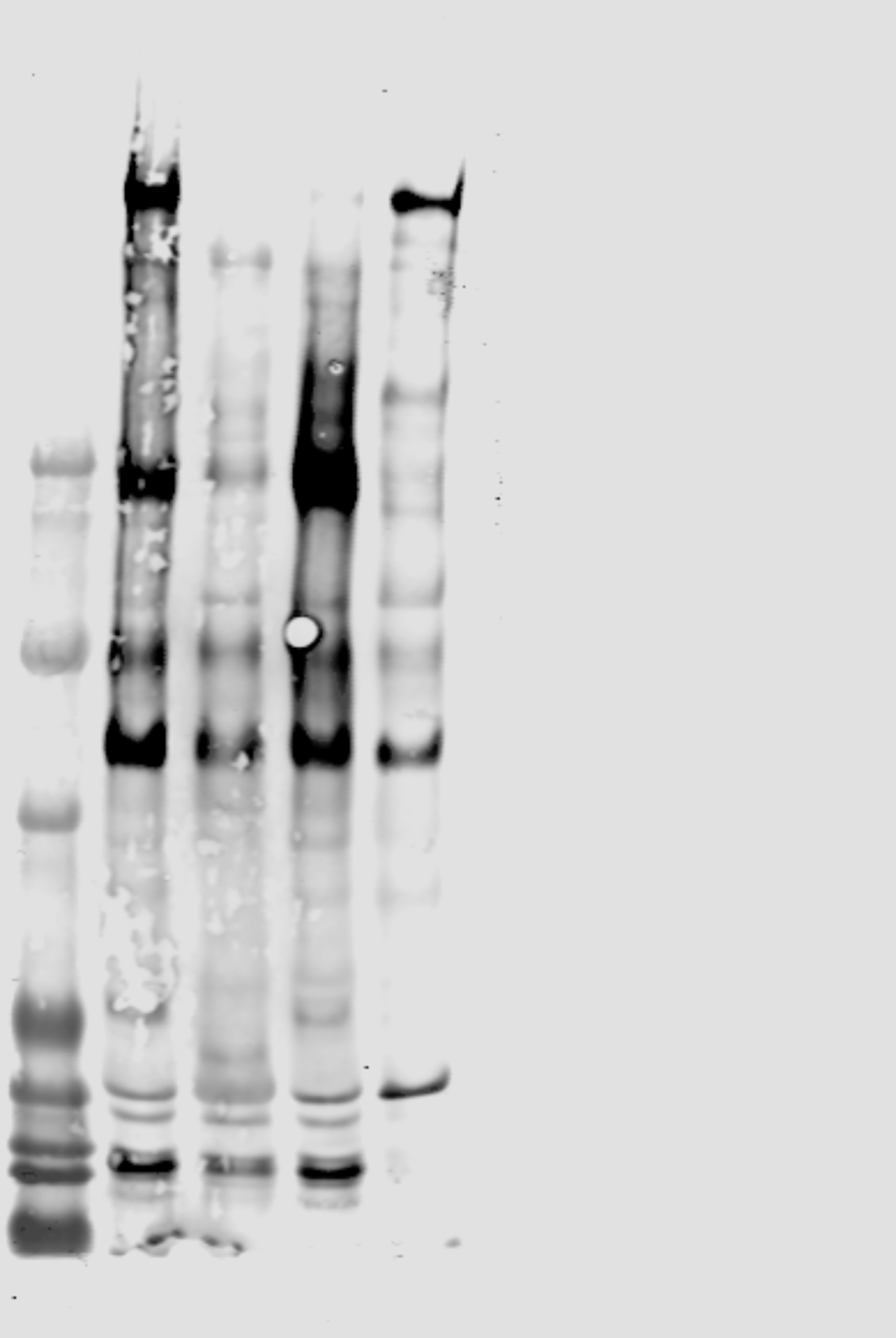

Supplement: Figure 1—source data 1. [file elife-69815-fig1-data1.zip › Figure 1 and Figure 1-Figure Supp 1-4-source data/Figure 1-source data 1.tif]

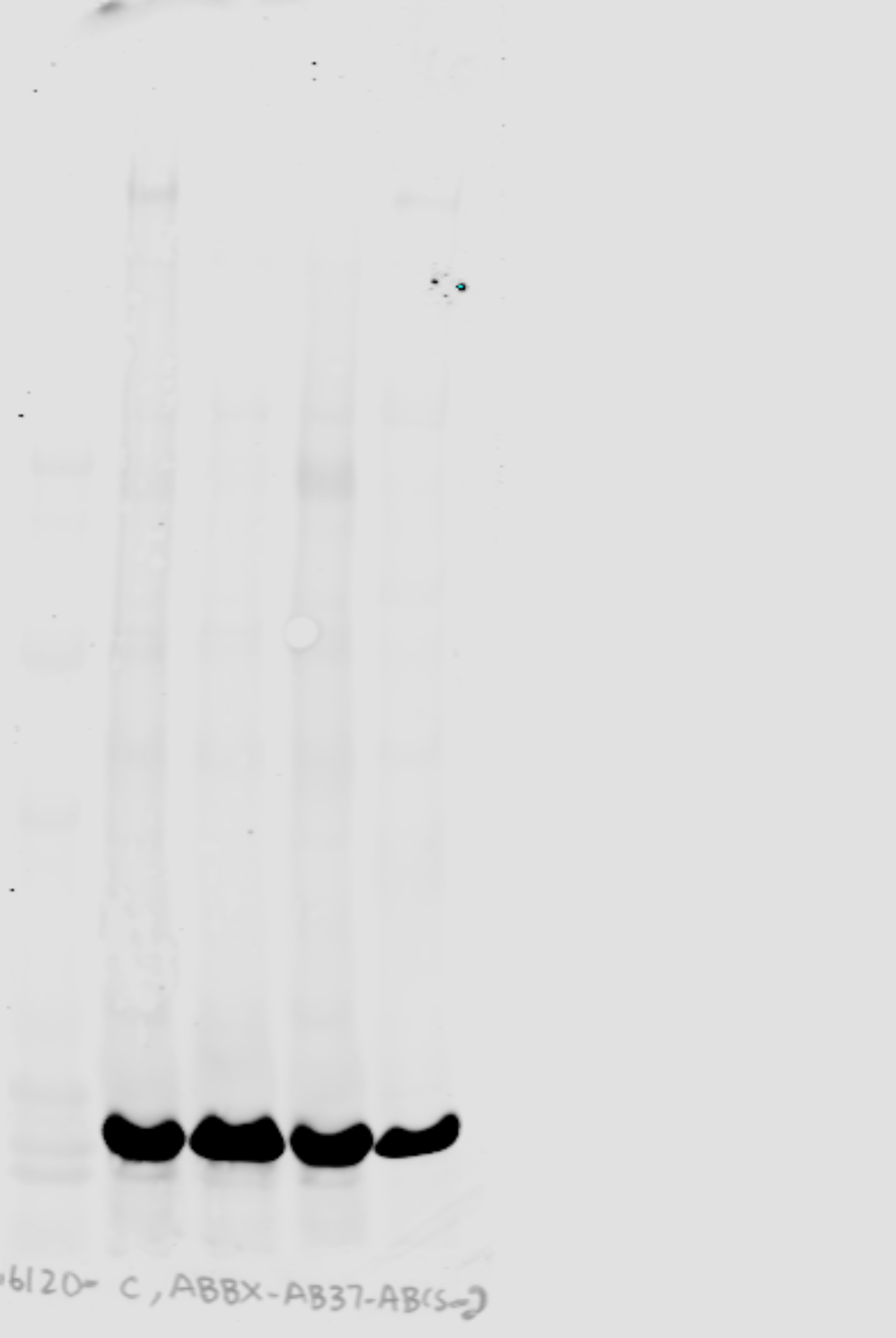

Supplement: Figure 1—source data 1. [file elife-69815-fig1-data1.zip › Figure 1 and Figure 1-Figure Supp 1-4-source data/Figure 1-source data 2.tif]

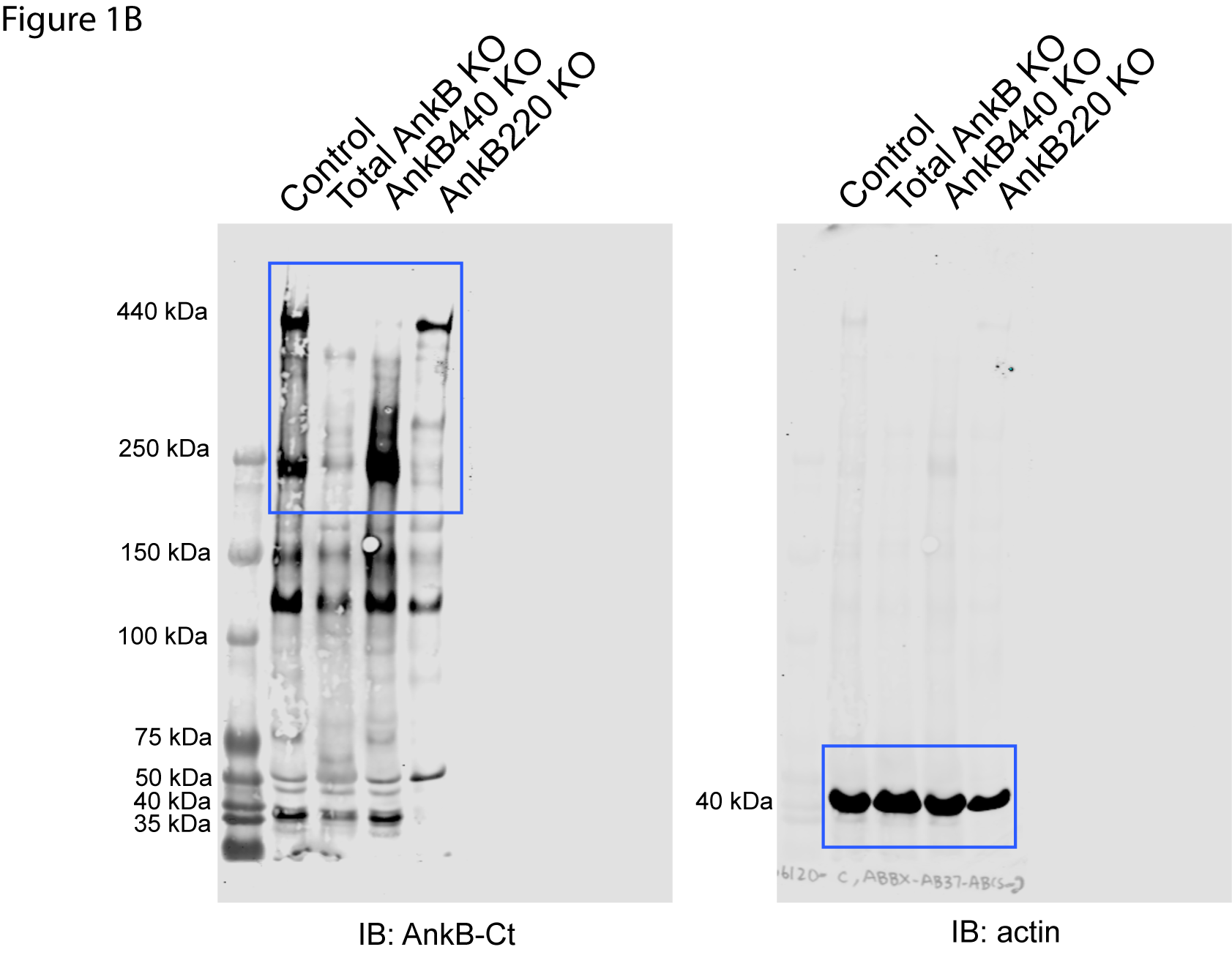

Supplement: Figure 1—source data 1. [file elife-69815-fig1-data1.zip › Figure 1 and Figure 1-Figure Supp 1-4-source data/Figure 1-source data 3.tif]

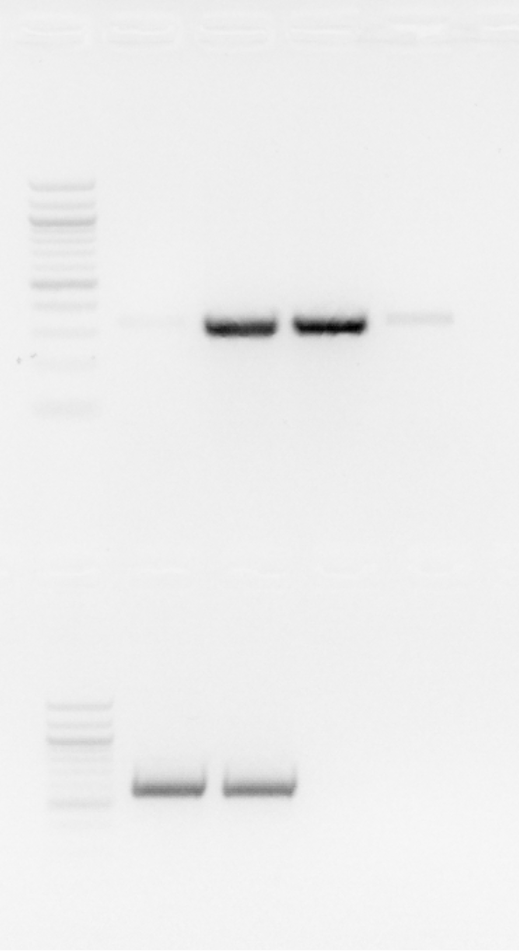

Supplement: Figure 1—source data 1. [file elife-69815-fig1-data1.zip › Figure 1 and Figure 1-Figure Supp 1-4-source data/Figure 1—figure supplement 1-source data 1.tif]

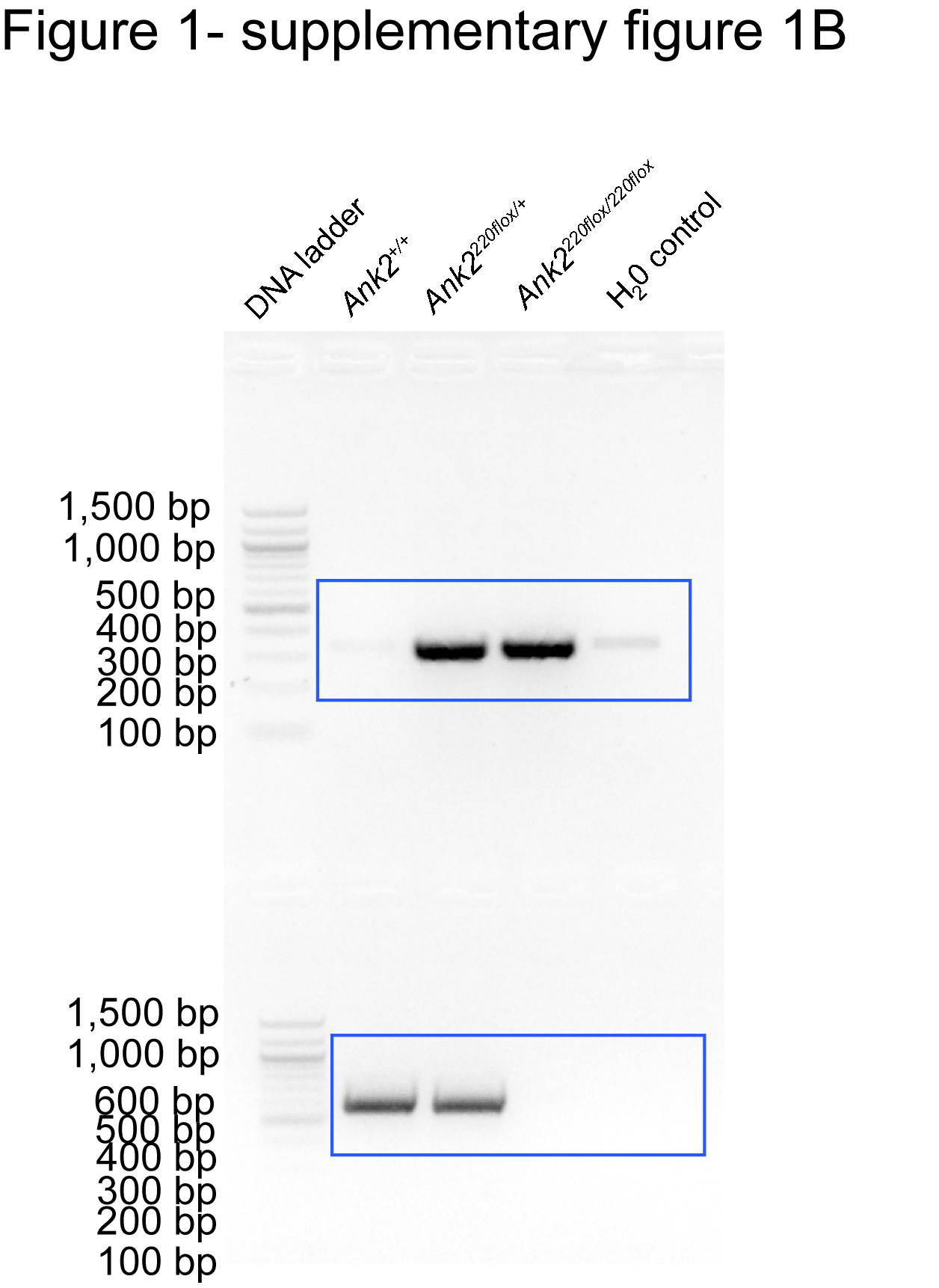

Supplement: Figure 1—source data 1. [file elife-69815-fig1-data1.zip › Figure 1 and Figure 1-Figure Supp 1-4-source data/Figure 1—figure supplement 1-source data 2.tif]

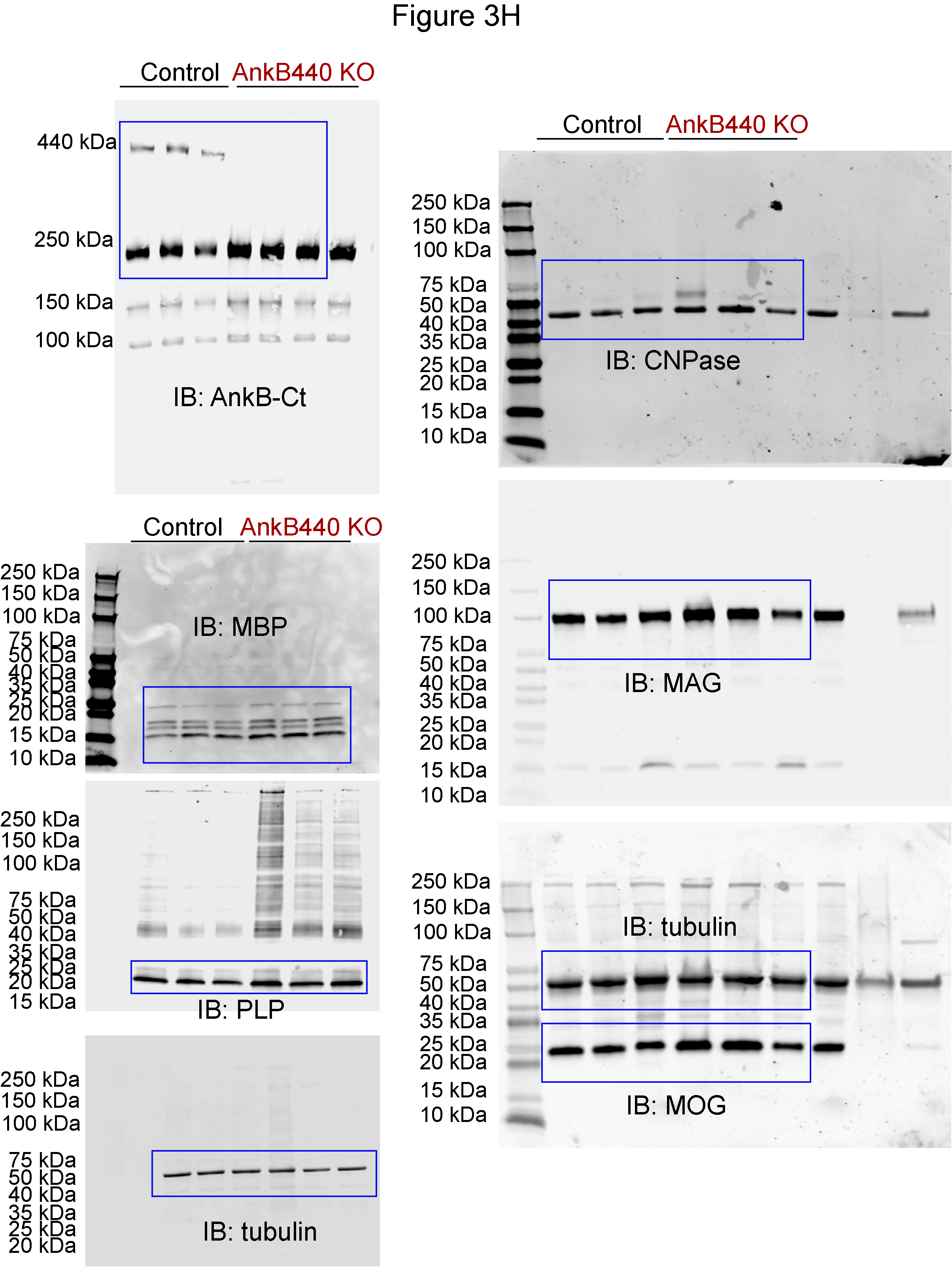

Supplement: Figure 3—source data 1. [file elife-69815-fig3-data1.zip › Figure 3 and Figure 3-Figure Supp 1 and 2-source data/Figure 3-souce data 8.tif]

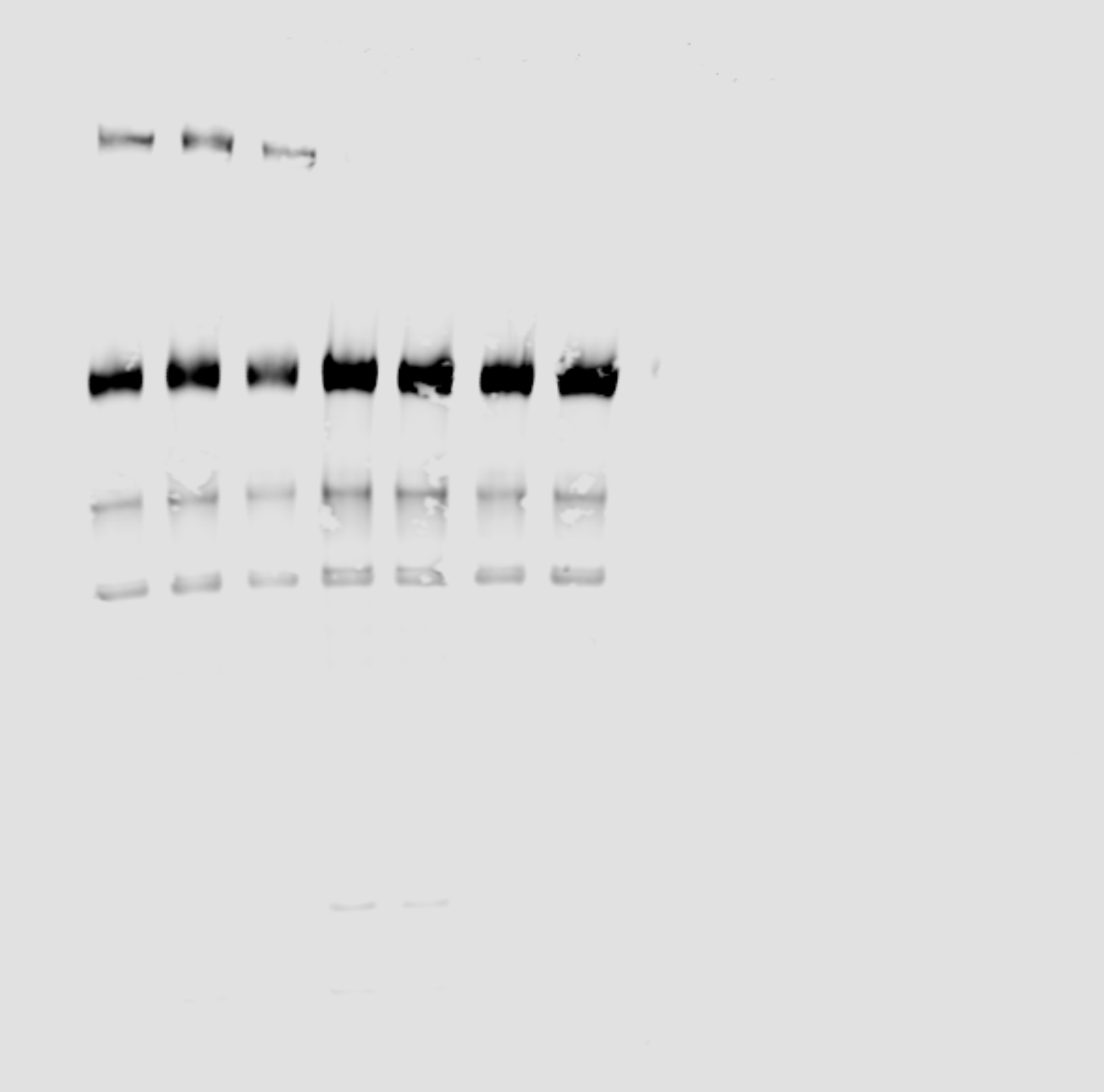

Supplement: Figure 3—source data 1. [file elife-69815-fig3-data1.zip › Figure 3 and Figure 3-Figure Supp 1 and 2-source data/Figure 3-source data 1.tif]

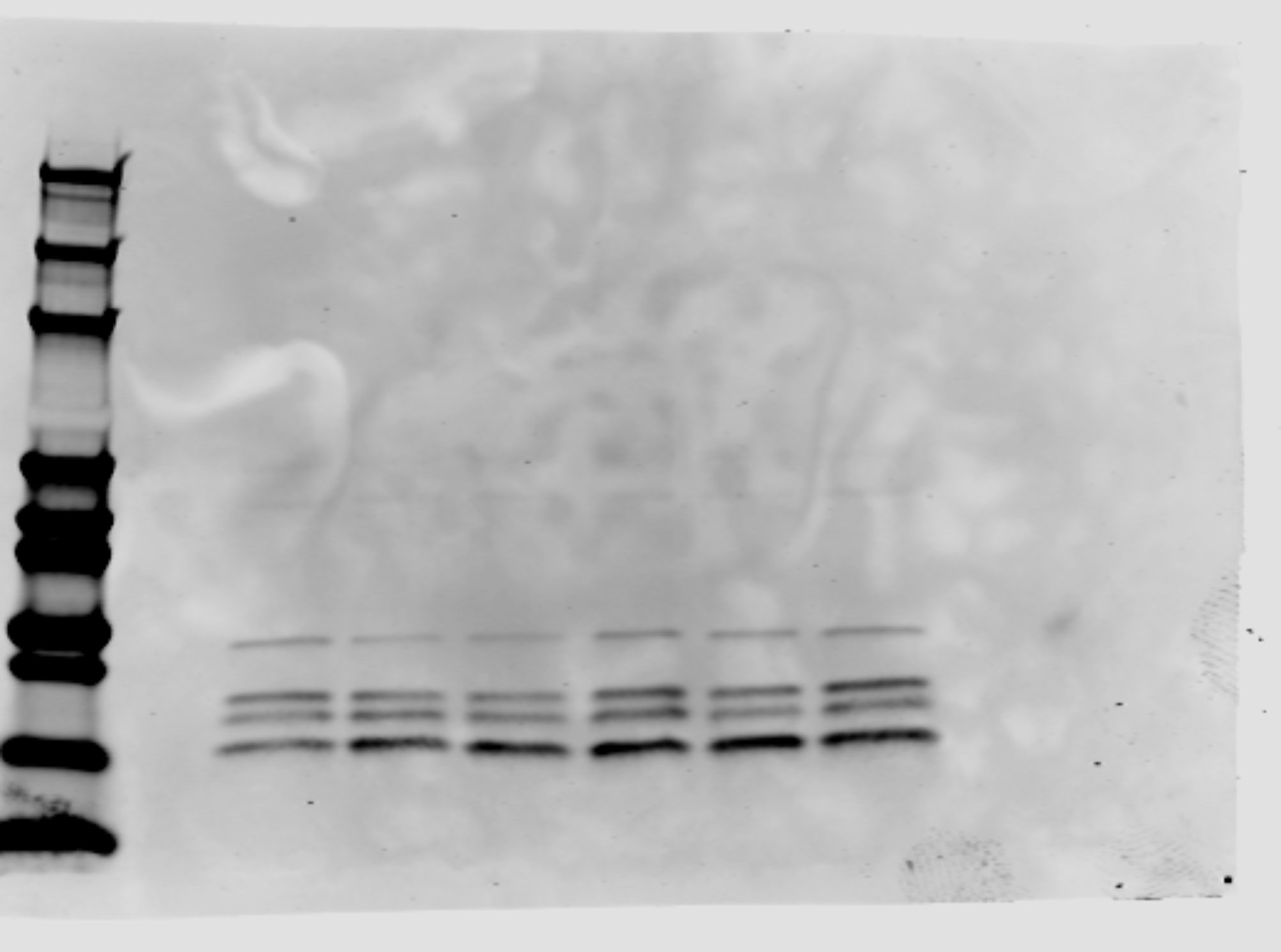

Supplement: Figure 3—source data 1. [file elife-69815-fig3-data1.zip › Figure 3 and Figure 3-Figure Supp 1 and 2-source data/Figure 3-source data 2.tif]

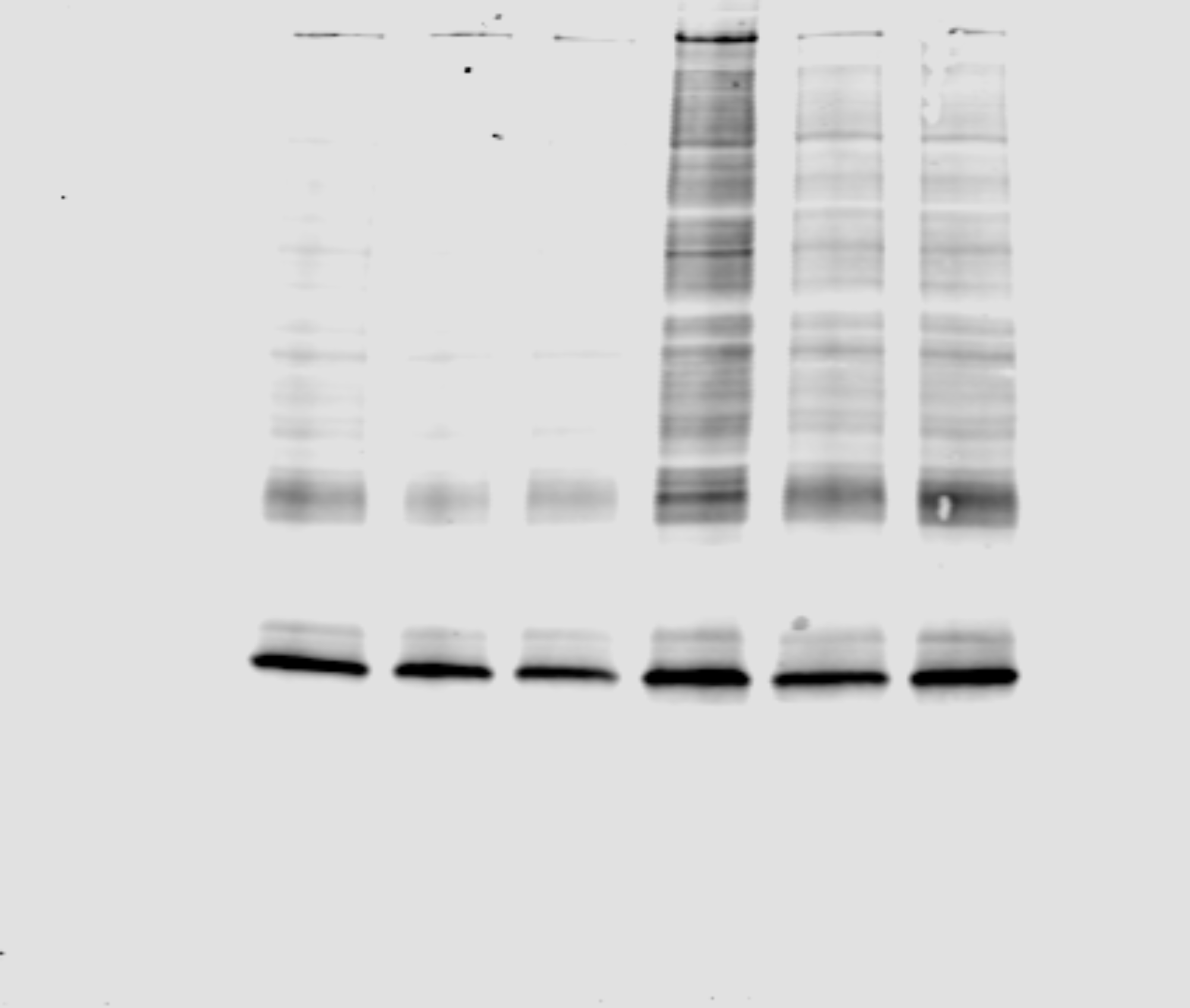

Supplement: Figure 3—source data 1. [file elife-69815-fig3-data1.zip › Figure 3 and Figure 3-Figure Supp 1 and 2-source data/Figure 3-source data 3.tif]

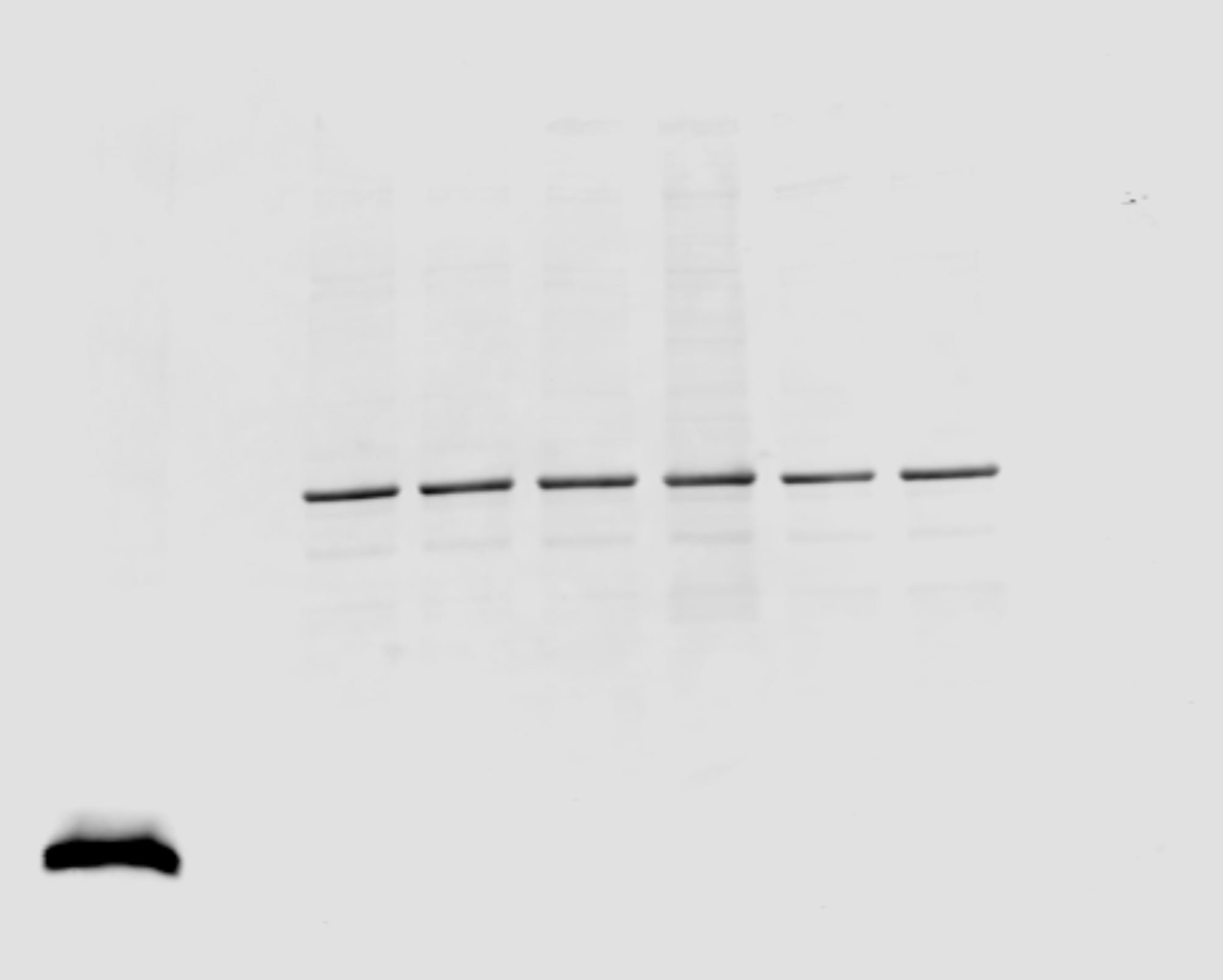

Supplement: Figure 3—source data 1. [file elife-69815-fig3-data1.zip › Figure 3 and Figure 3-Figure Supp 1 and 2-source data/Figure 3-source data 4.tif]

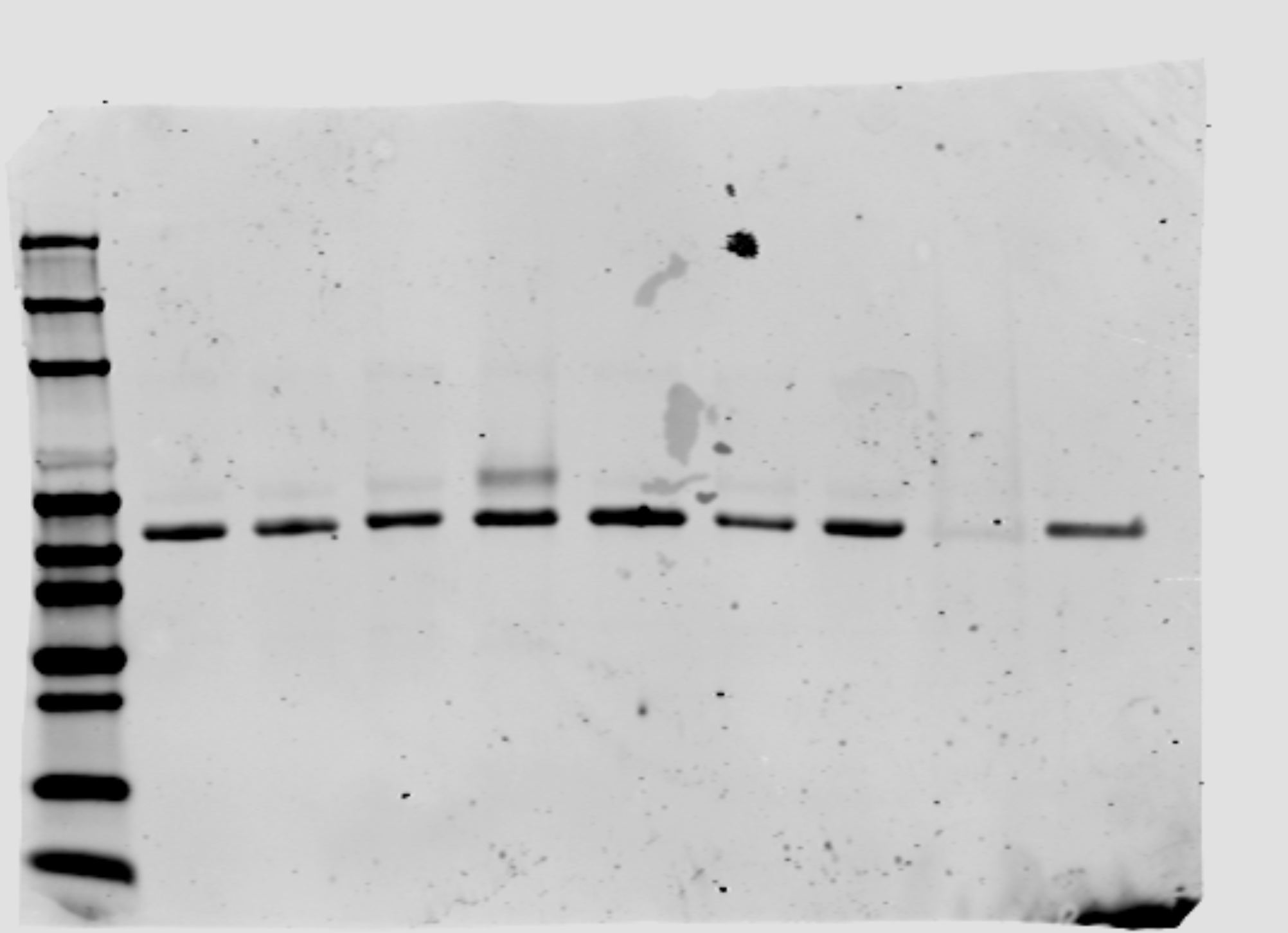

Supplement: Figure 3—source data 1. [file elife-69815-fig3-data1.zip › Figure 3 and Figure 3-Figure Supp 1 and 2-source data/Figure 3-source data 5.tif]

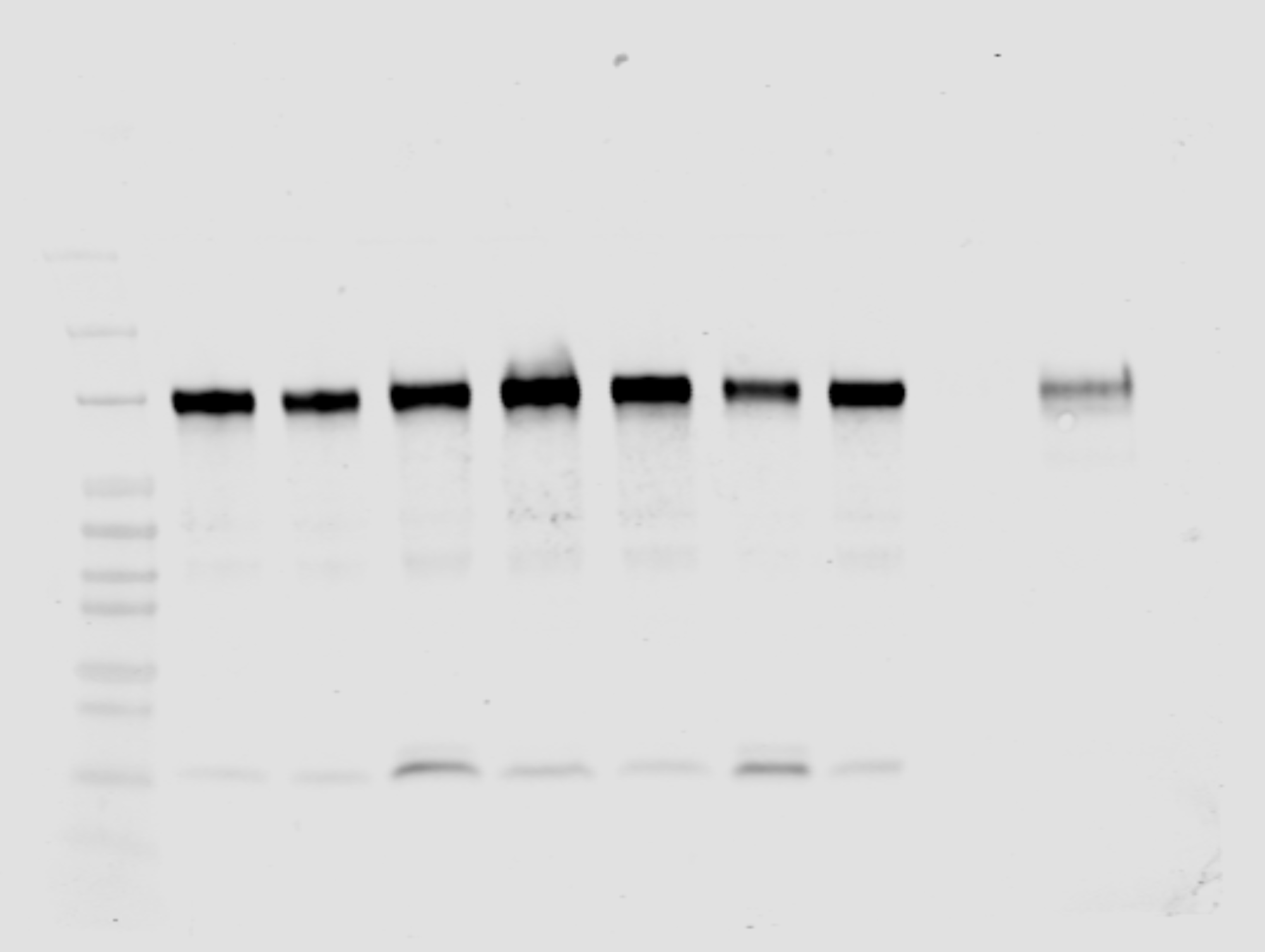

Supplement: Figure 3—source data 1. [file elife-69815-fig3-data1.zip › Figure 3 and Figure 3-Figure Supp 1 and 2-source data/Figure 3-source data 6.tif]

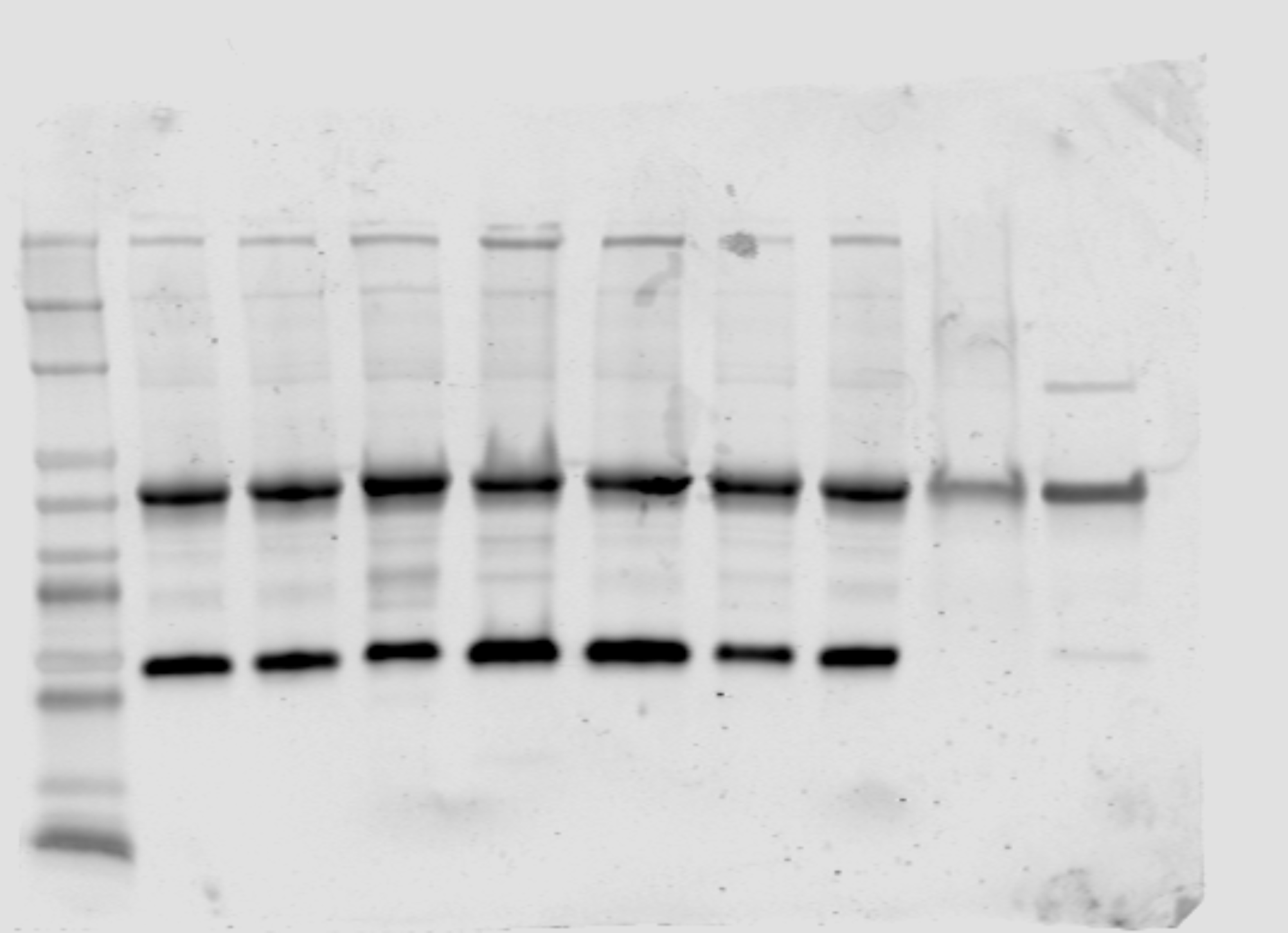

Supplement: Figure 3—source data 1. [file elife-69815-fig3-data1.zip › Figure 3 and Figure 3-Figure Supp 1 and 2-source data/Figure 3-source data 7.tif]

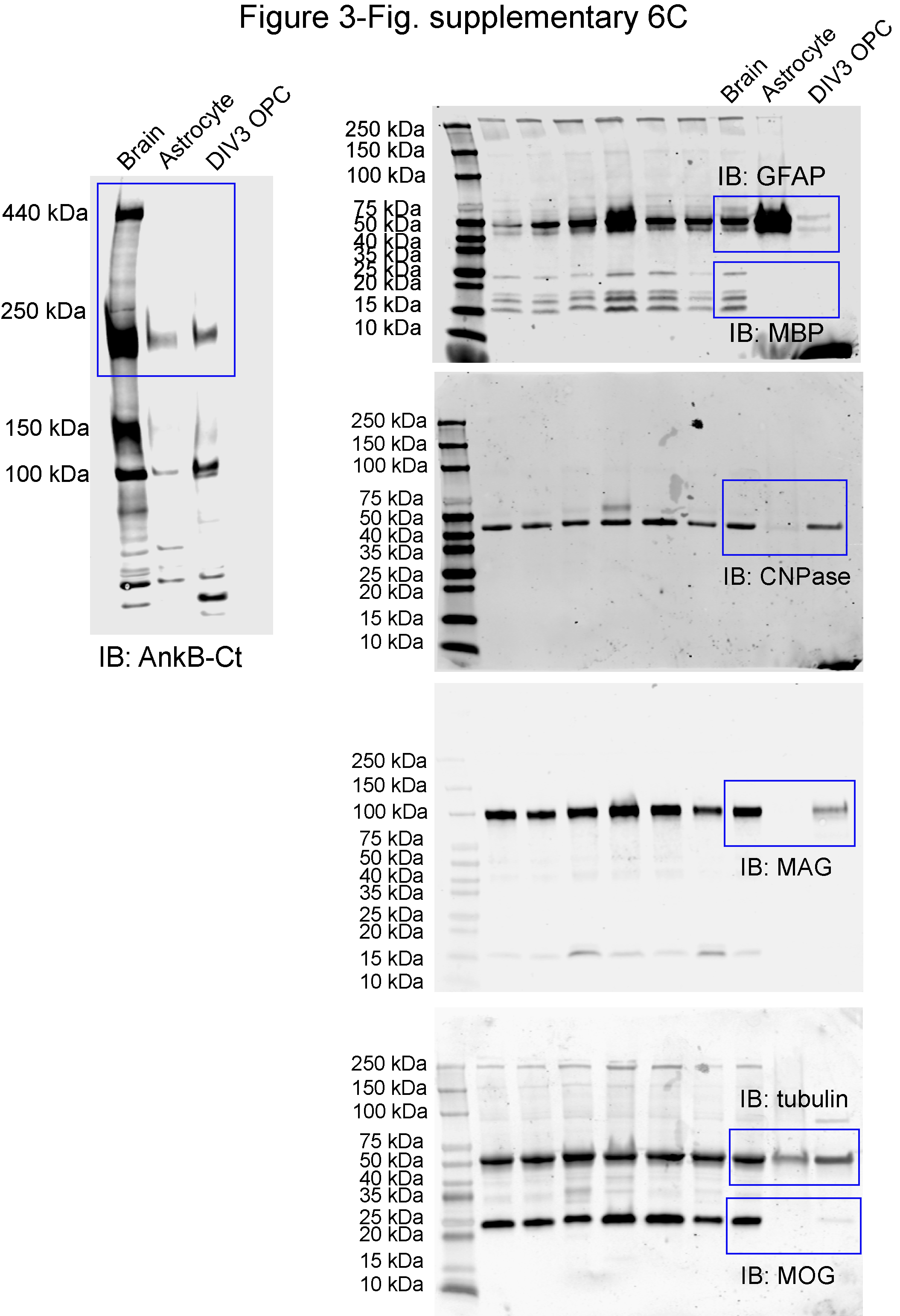

Supplement: Figure 3—source data 1. [file elife-69815-fig3-data1.zip › Figure 3 and Figure 3-Figure Supp 1 and 2-source data/Figure 3—figure supplement 1-souce data 6.tif]

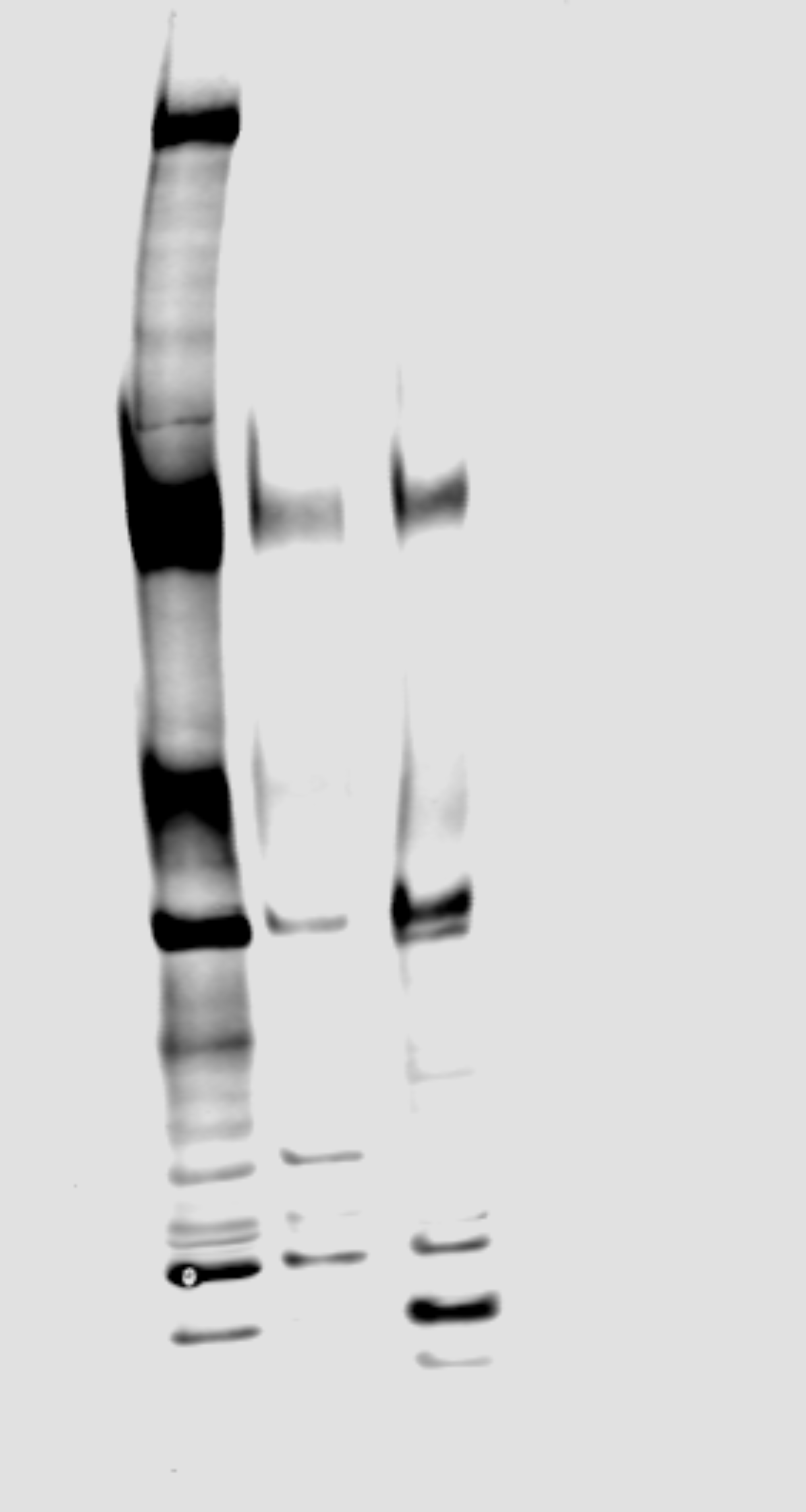

Supplement: Figure 3—source data 1. [file elife-69815-fig3-data1.zip › Figure 3 and Figure 3-Figure Supp 1 and 2-source data/Figure 3—figure supplement 1-source data 1.tif]

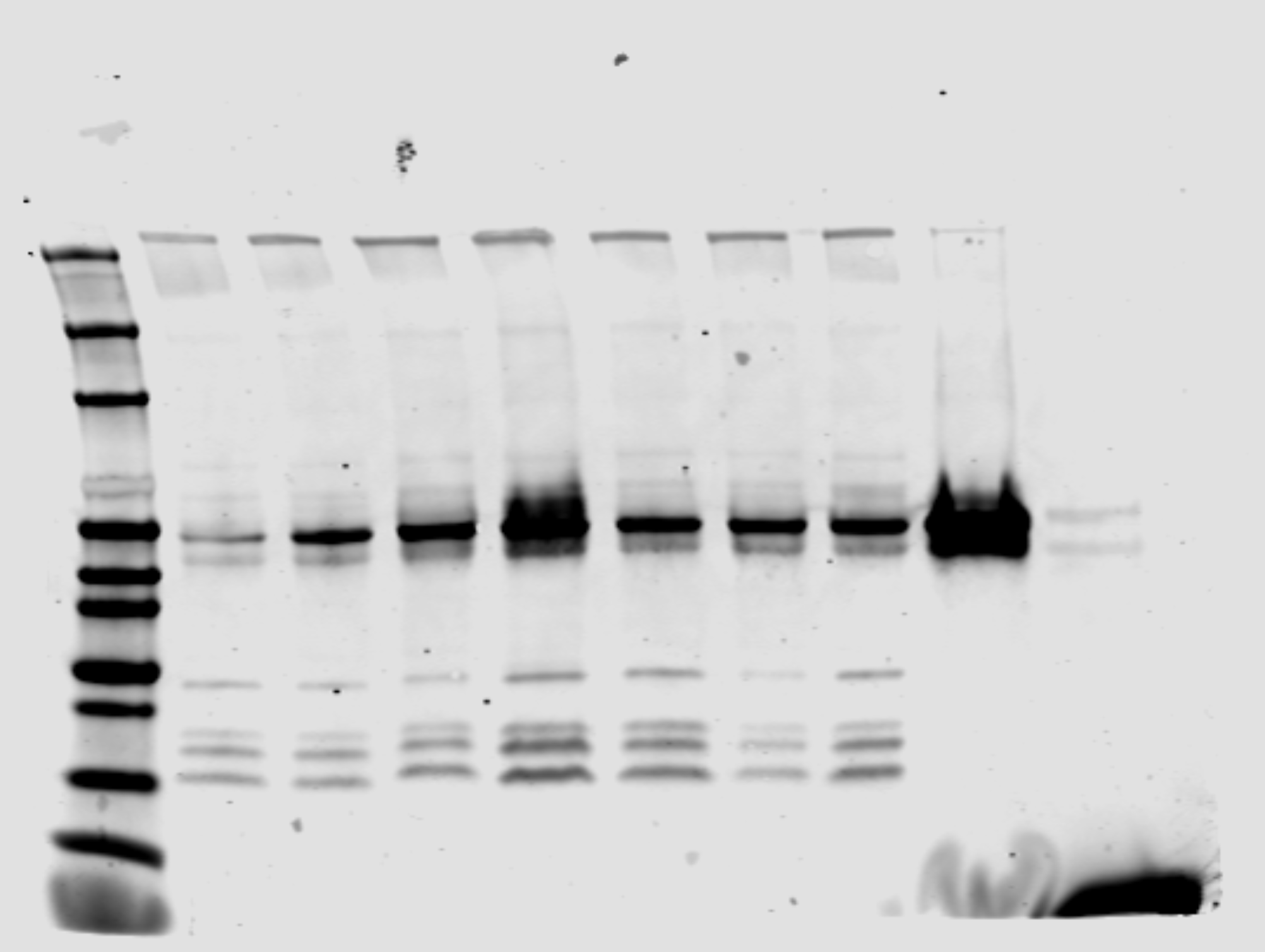

Supplement: Figure 3—source data 1. [file elife-69815-fig3-data1.zip › Figure 3 and Figure 3-Figure Supp 1 and 2-source data/Figure 3—figure supplement 1-source data 2.tif]

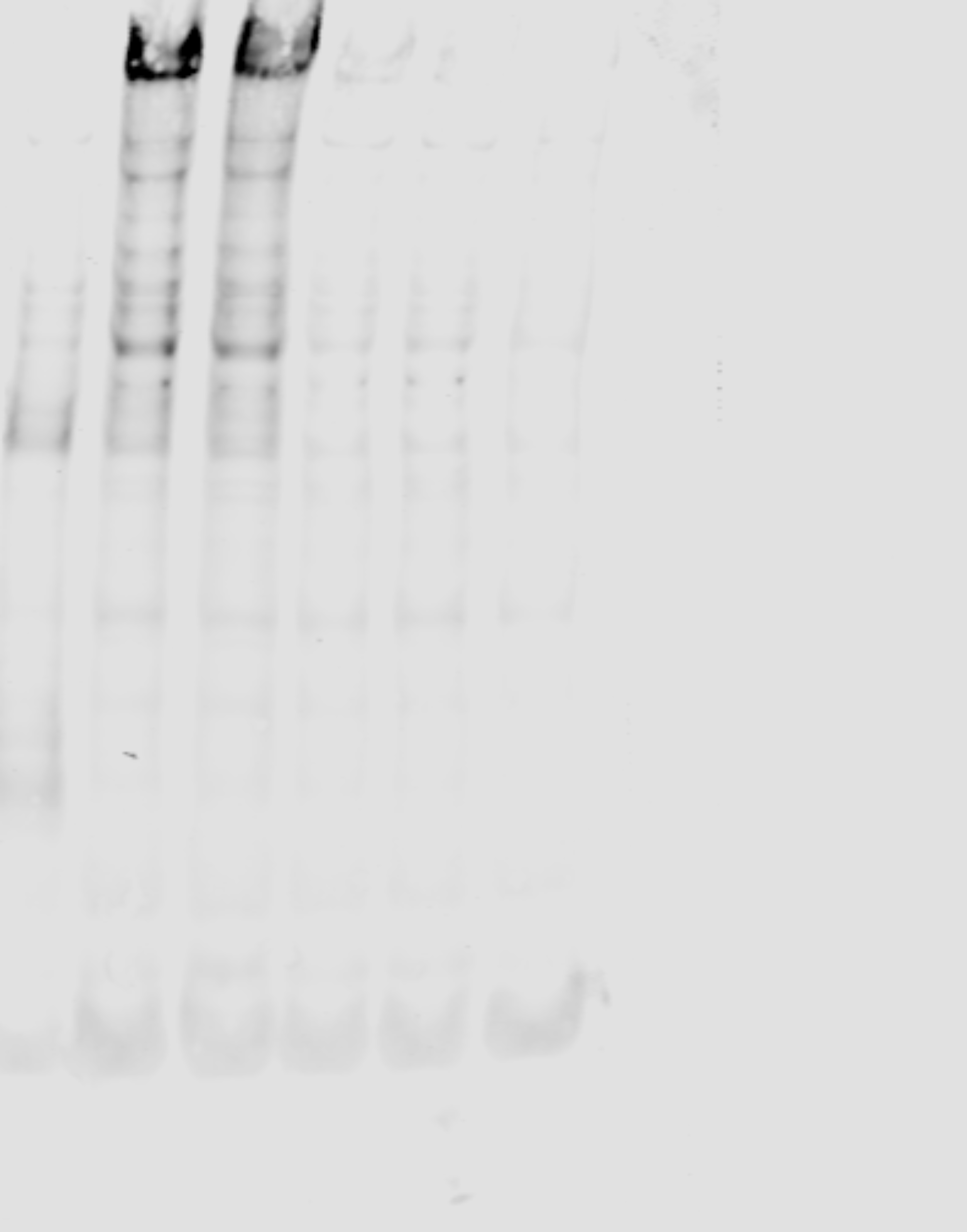

Supplement: Figure 5—source data 1. [file elife-69815-fig5-data1.zip › Figure 5 and Figure 5-Figure Supp1-source data/Figure 5—figure supplement 1-source data 1.tif]

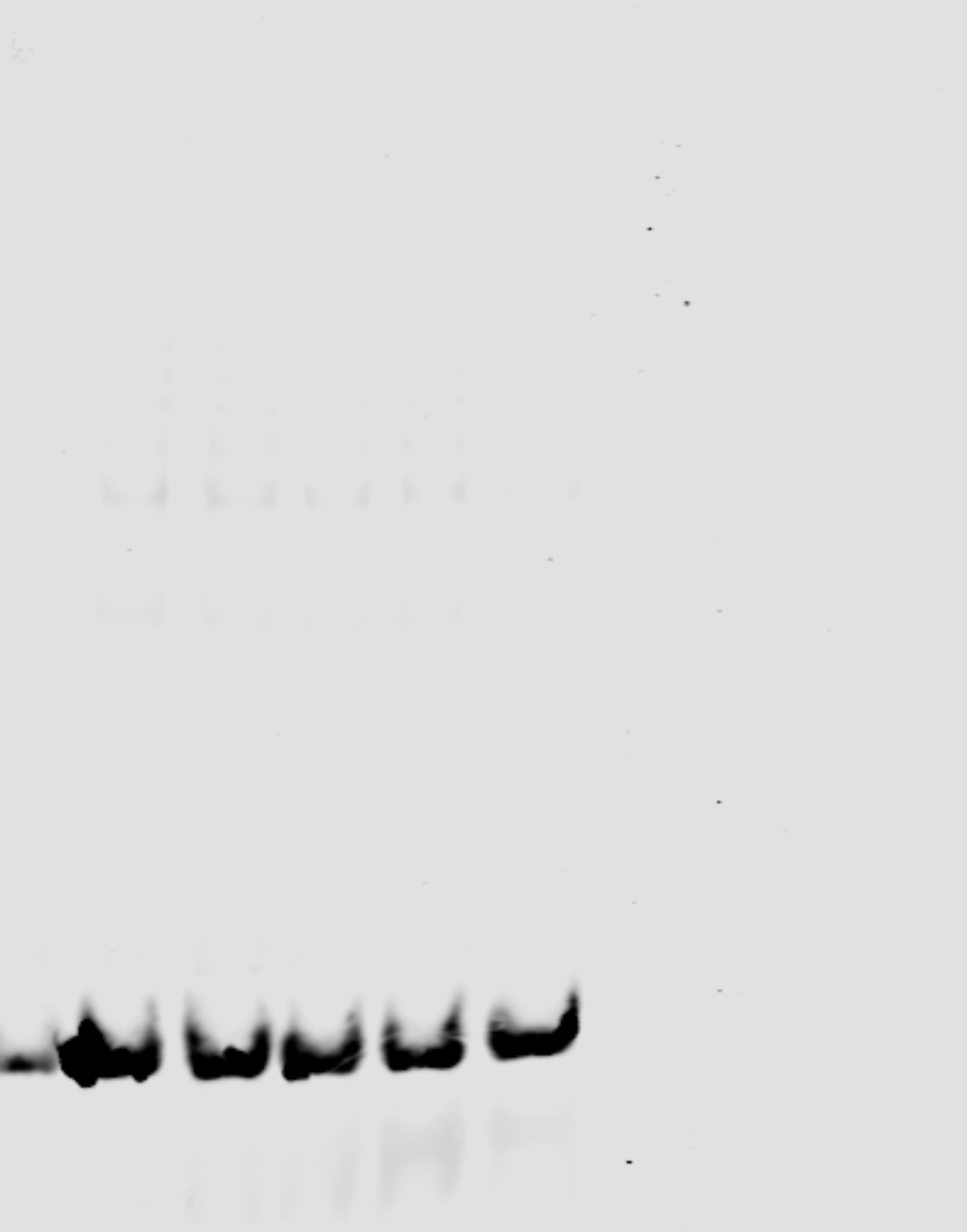

Supplement: Figure 5—source data 1. [file elife-69815-fig5-data1.zip › Figure 5 and Figure 5-Figure Supp1-source data/Figure 5—figure supplement 1-source data 2.tif]

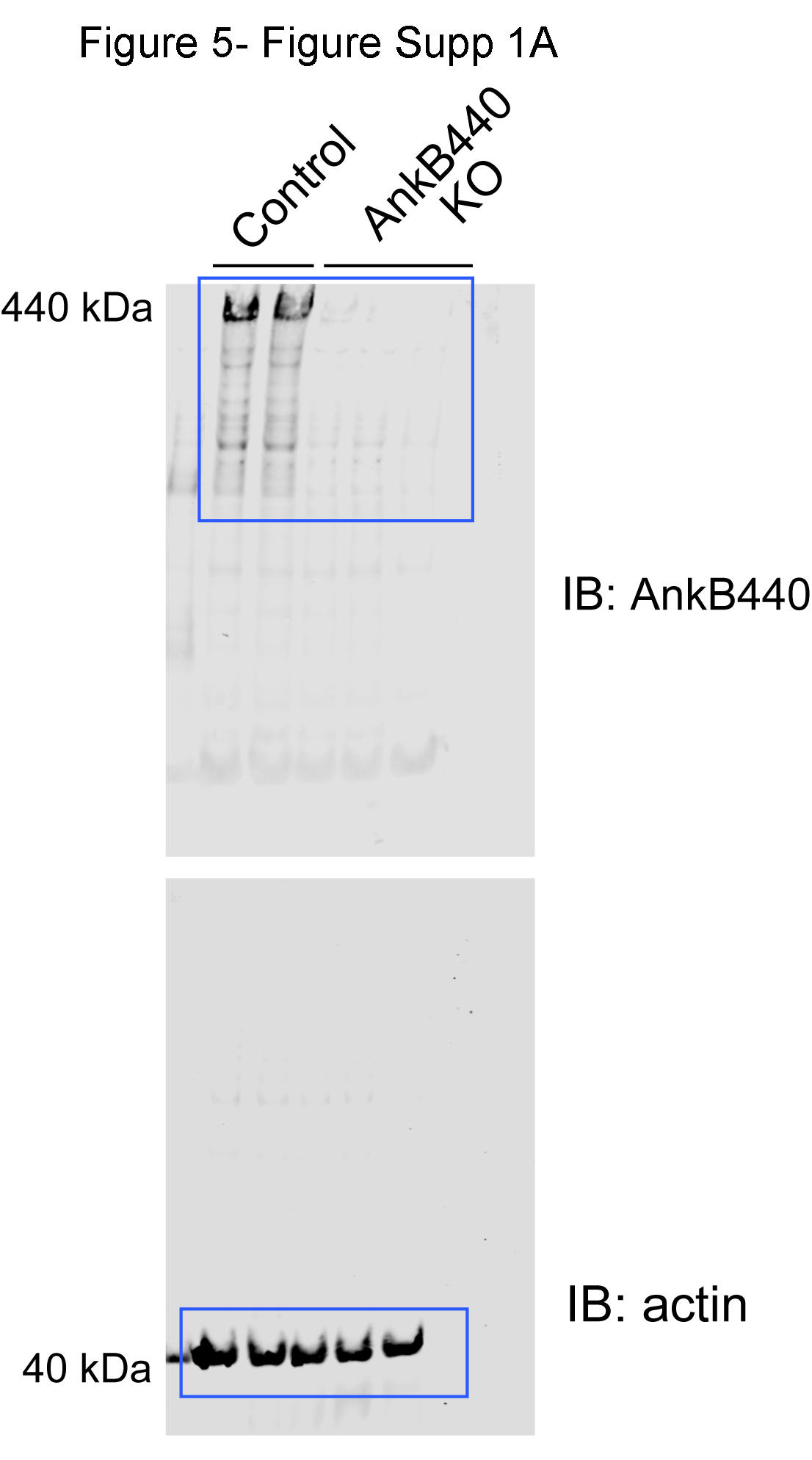

Supplement: Figure 5—source data 1. [file elife-69815-fig5-data1.zip › Figure 5 and Figure 5-Figure Supp1-source data/Figure 5—figure supplement 1-source data 3.tif]

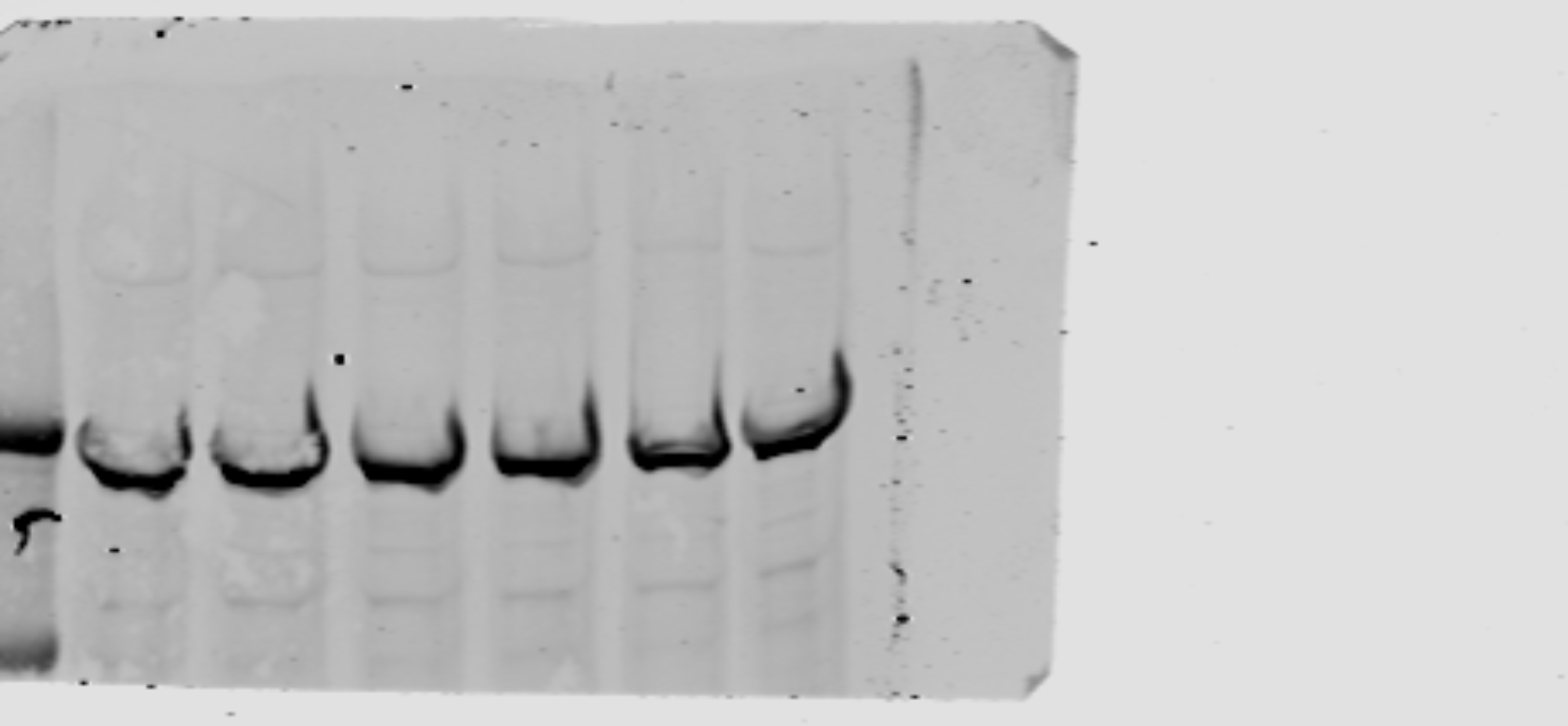

Supplement: Figure 7—source data 1. [file elife-69815-fig7-data1.zip › Figure 7 and Figure 7-Figure Supp 1-source data/Figure 7-source data 1.tif]

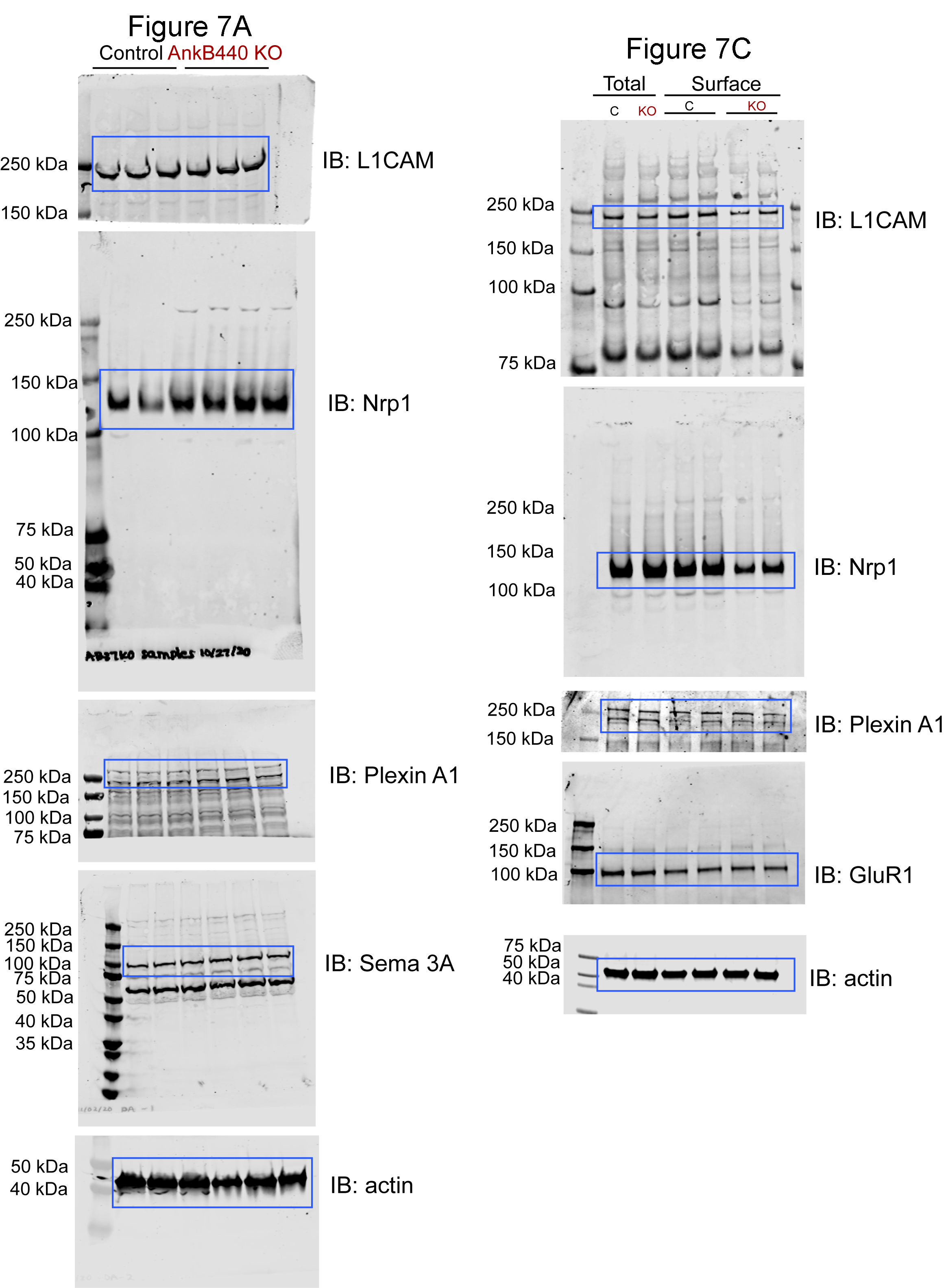

Supplement: Figure 7—source data 1. [file elife-69815-fig7-data1.zip › Figure 7 and Figure 7-Figure Supp 1-source data/Figure 7-source data 10.tif]

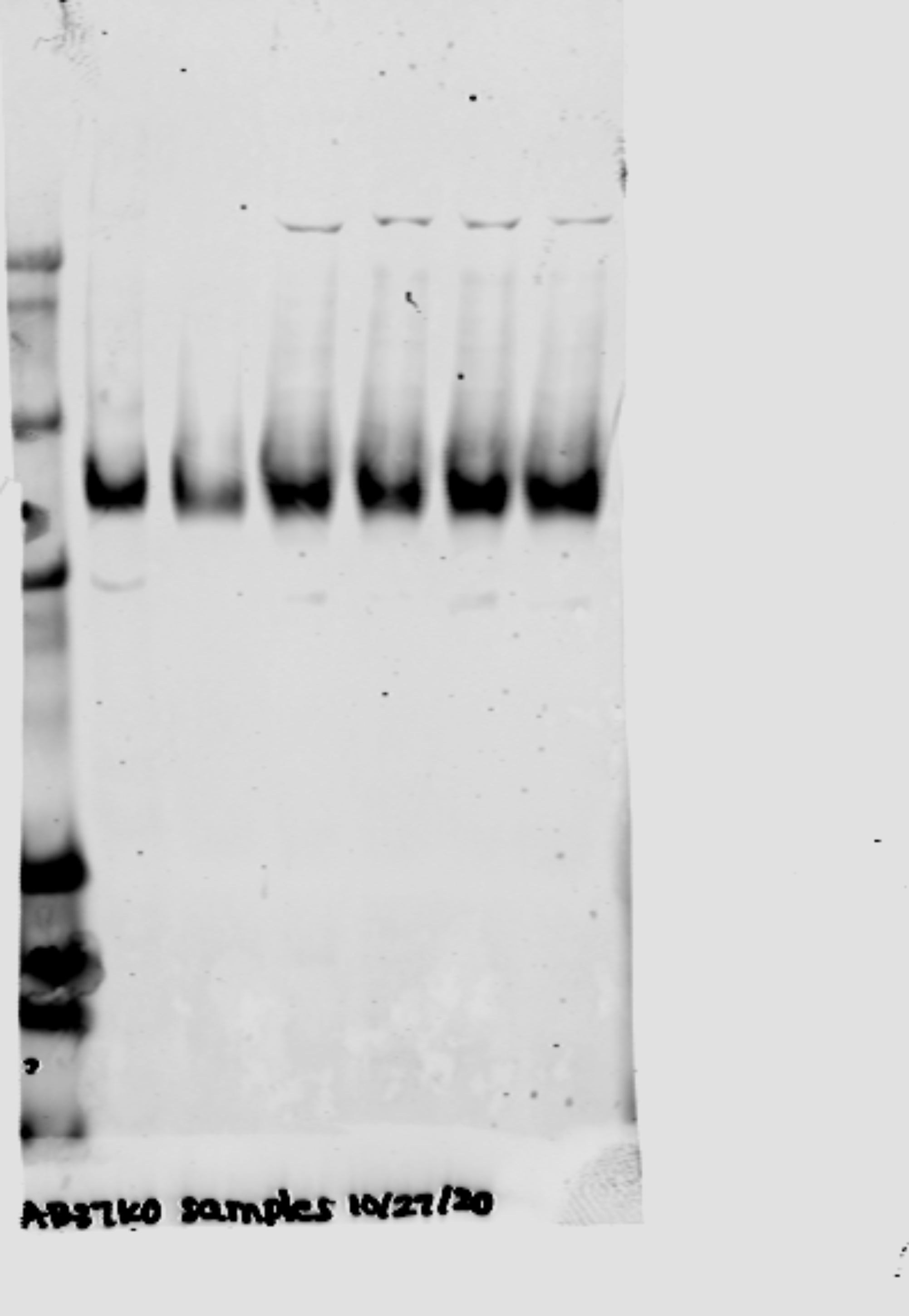

Supplement: Figure 7—source data 1. [file elife-69815-fig7-data1.zip › Figure 7 and Figure 7-Figure Supp 1-source data/Figure 7-source data 2.tif]

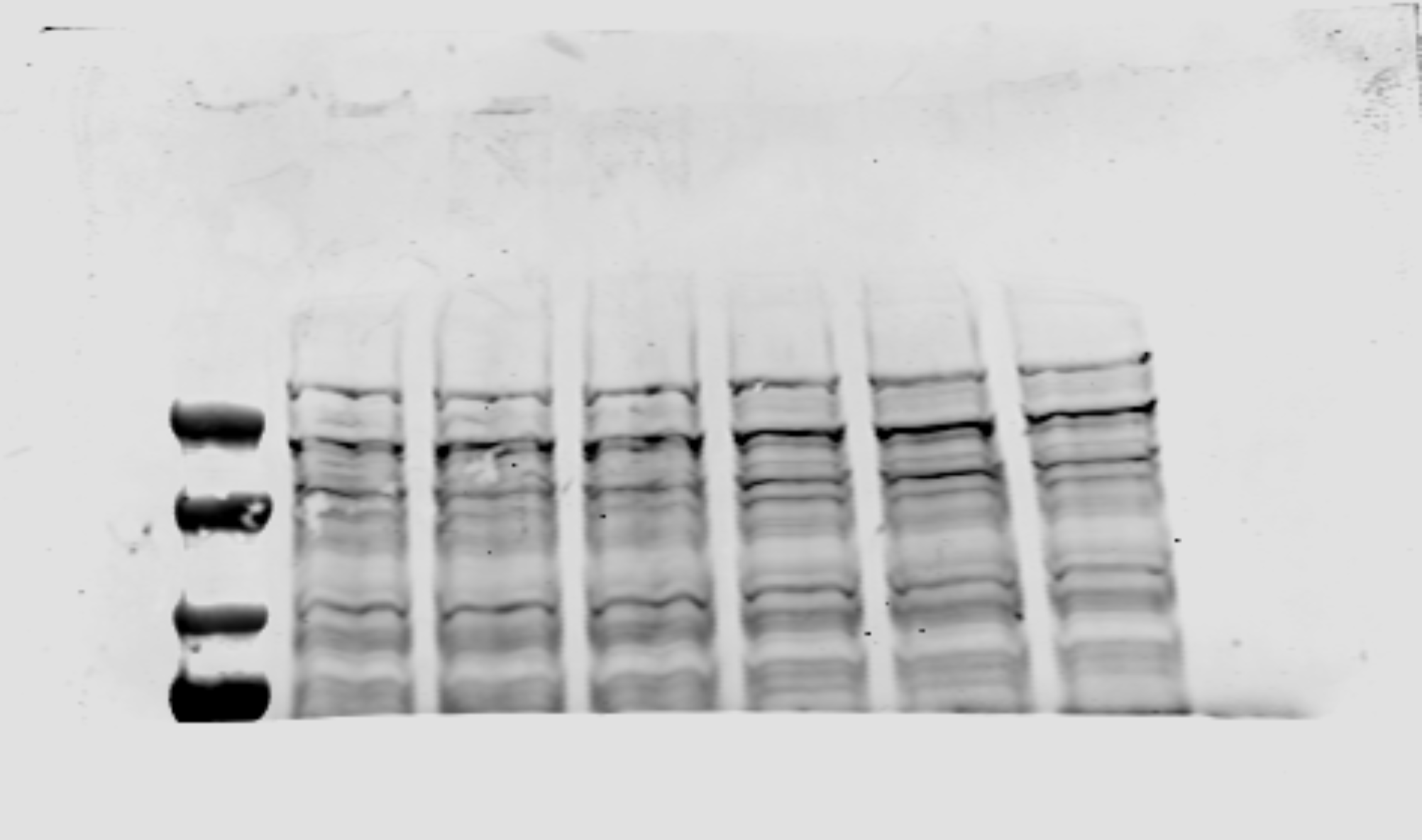

Supplement: Figure 7—source data 1. [file elife-69815-fig7-data1.zip › Figure 7 and Figure 7-Figure Supp 1-source data/Figure 7-source data 3.tif]

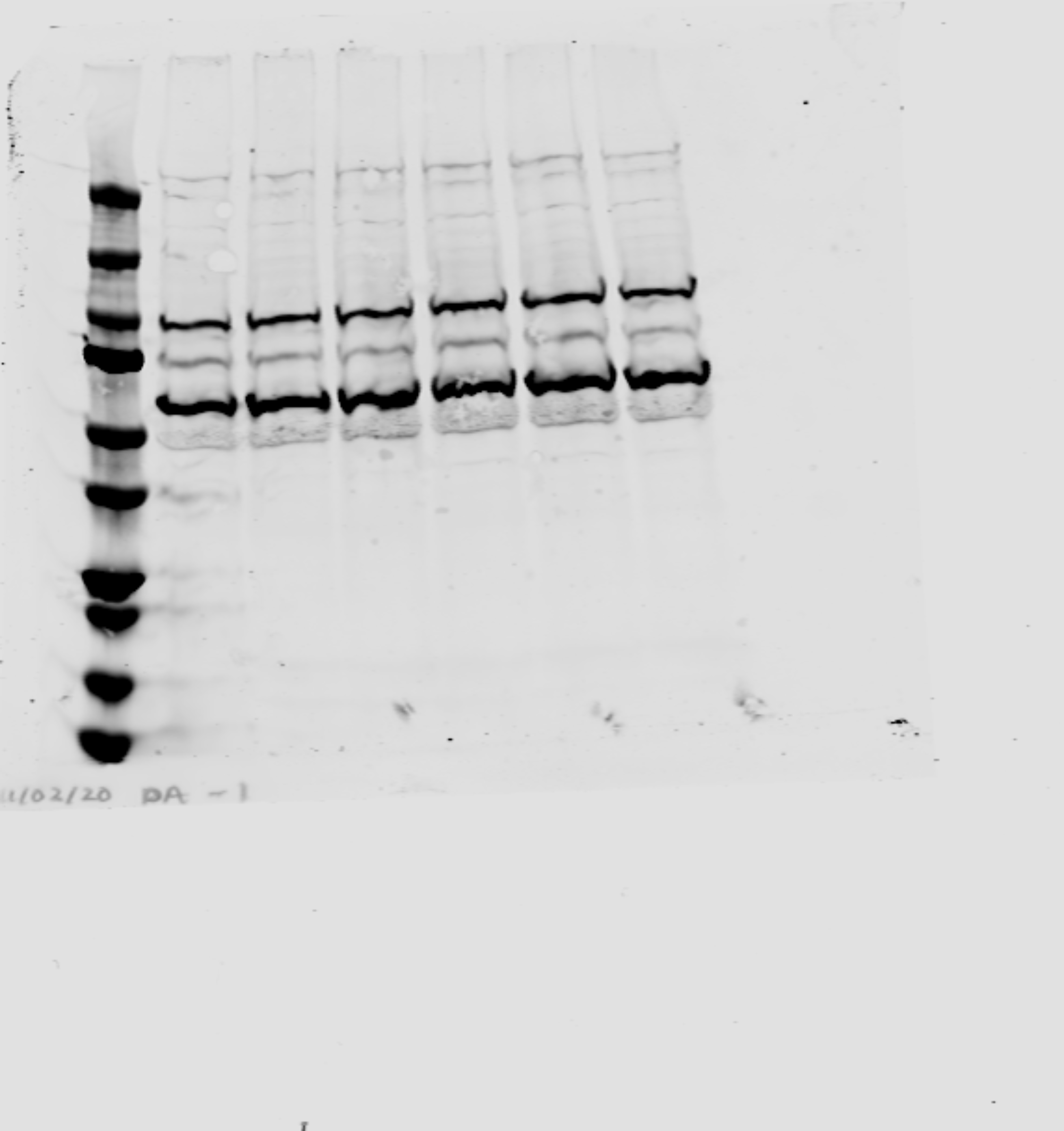

Supplement: Figure 7—source data 1. [file elife-69815-fig7-data1.zip › Figure 7 and Figure 7-Figure Supp 1-source data/Figure 7-source data 4.tif]

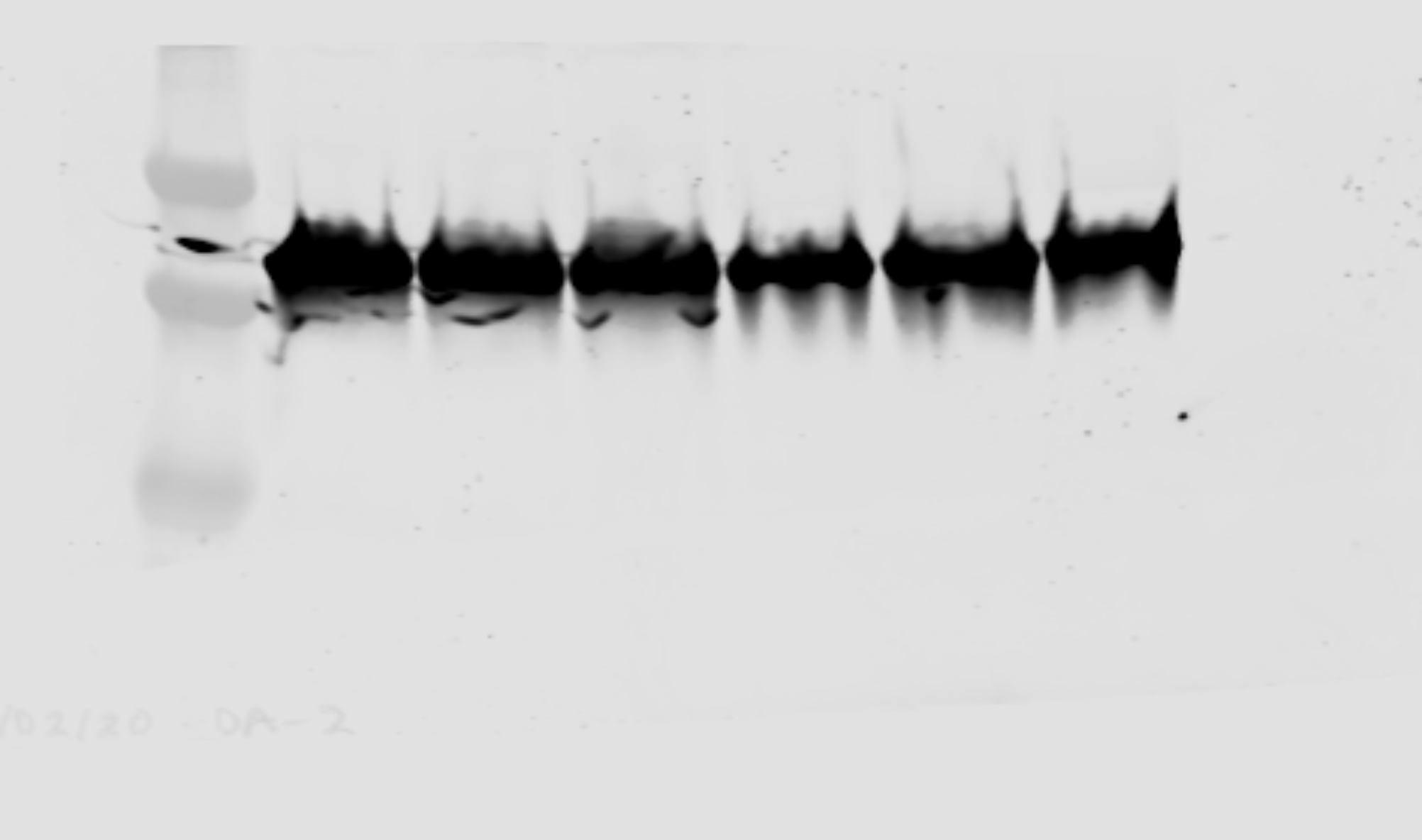

Supplement: Figure 7—source data 1. [file elife-69815-fig7-data1.zip › Figure 7 and Figure 7-Figure Supp 1-source data/Figure 7-source data 5.tif]

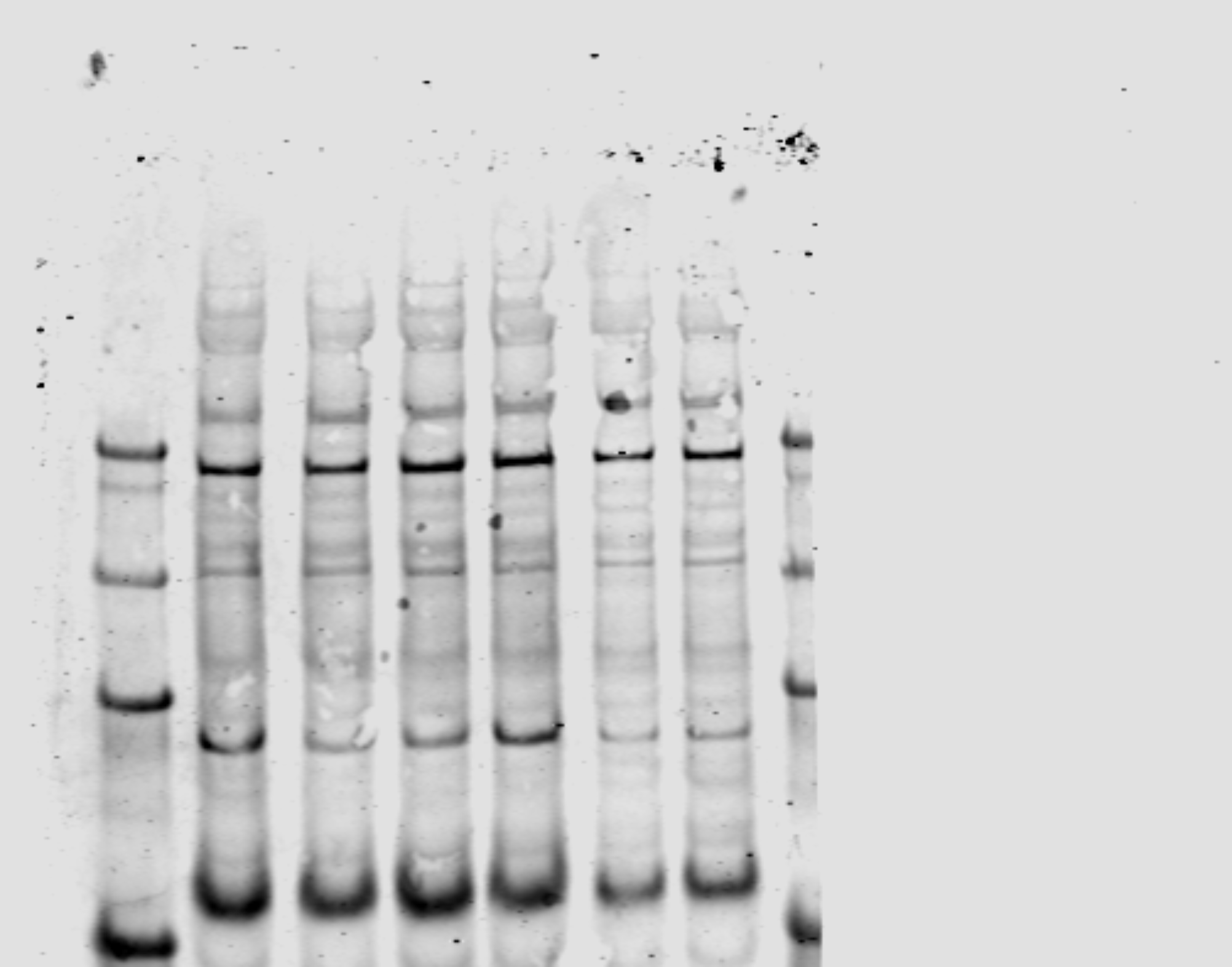

Supplement: Figure 7—source data 1. [file elife-69815-fig7-data1.zip › Figure 7 and Figure 7-Figure Supp 1-source data/Figure 7-source data 6.tif]

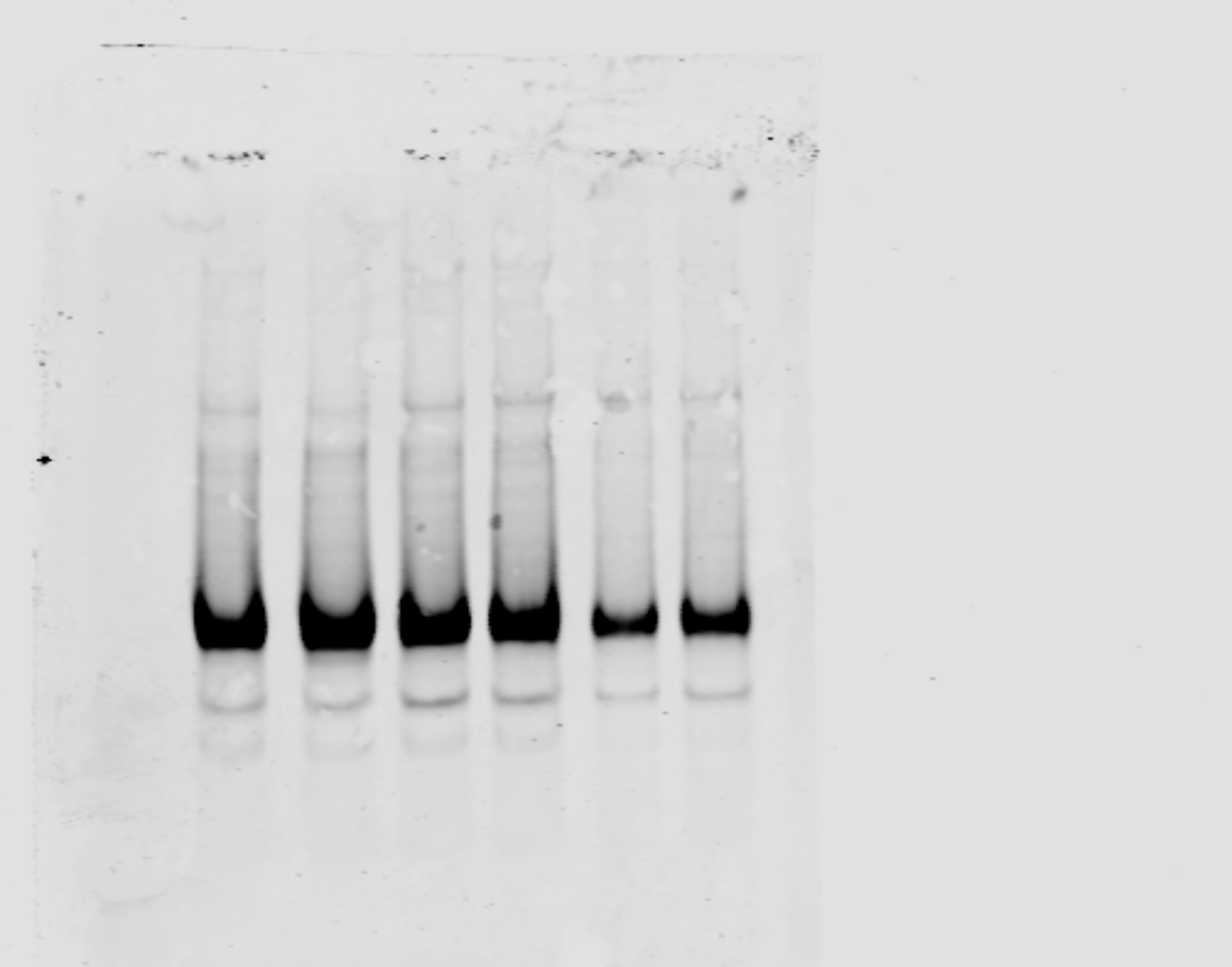

Supplement: Figure 7—source data 1. [file elife-69815-fig7-data1.zip › Figure 7 and Figure 7-Figure Supp 1-source data/Figure 7-source data 7.tif]

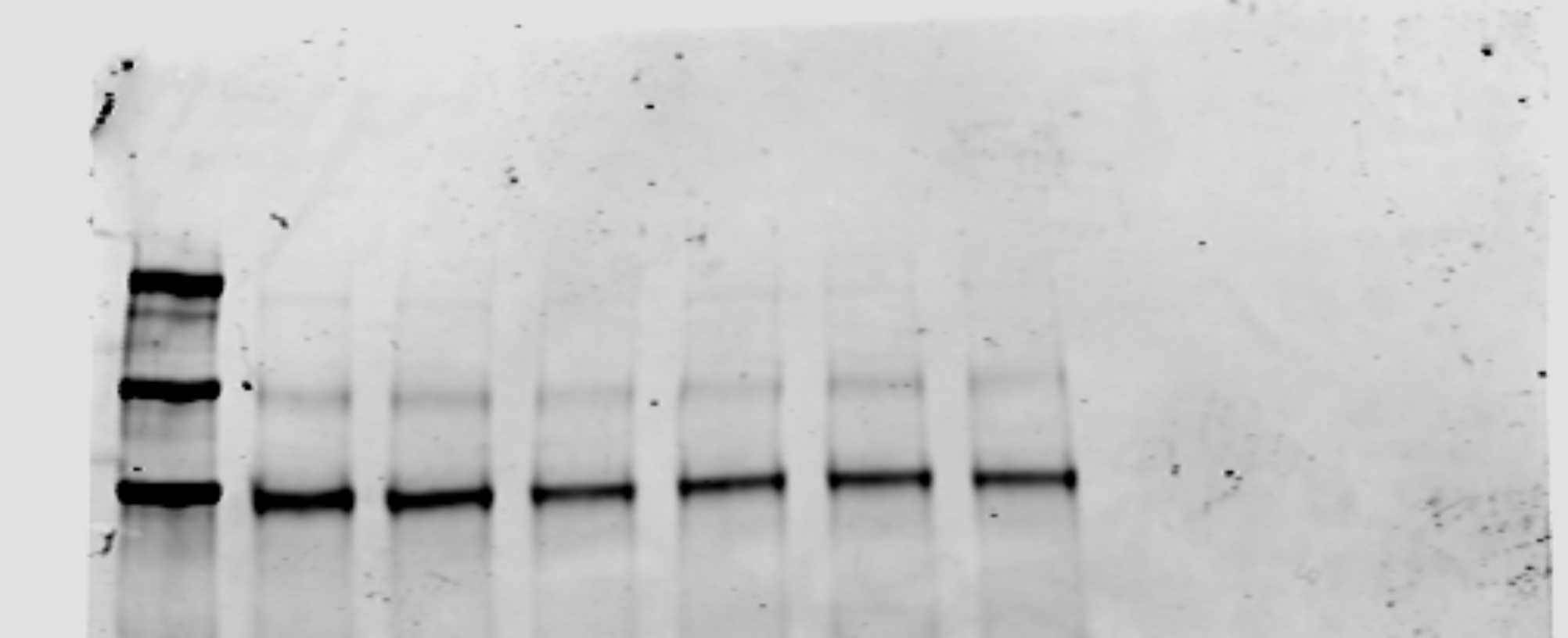

Supplement: Figure 7—source data 1. [file elife-69815-fig7-data1.zip › Figure 7 and Figure 7-Figure Supp 1-source data/Figure 7-source data 8.tif]

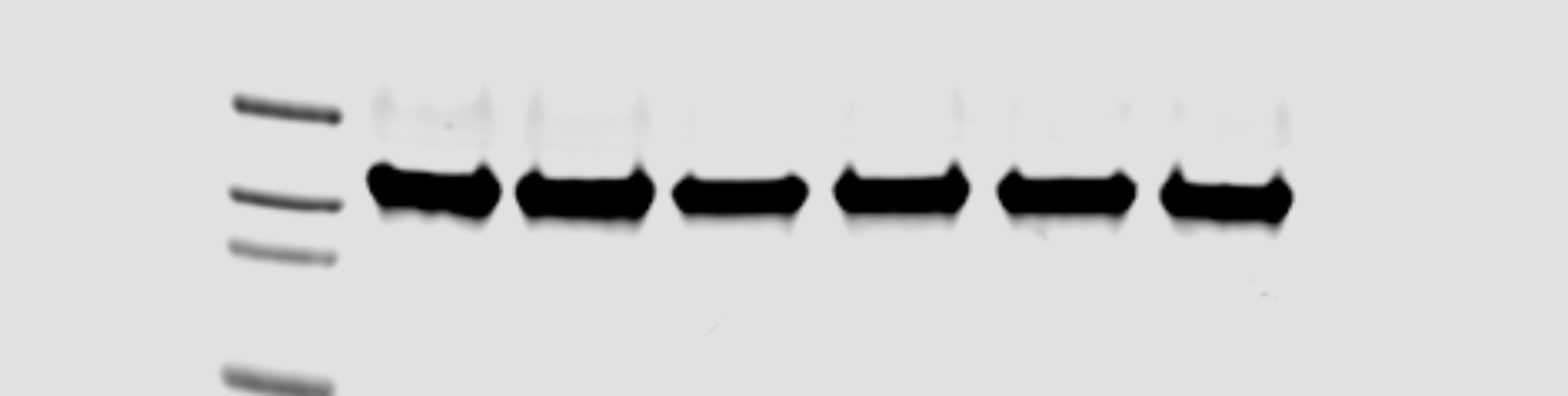

Supplement: Figure 7—source data 1. [file elife-69815-fig7-data1.zip › Figure 7 and Figure 7-Figure Supp 1-source data/Figure 7-source data 9.tif]

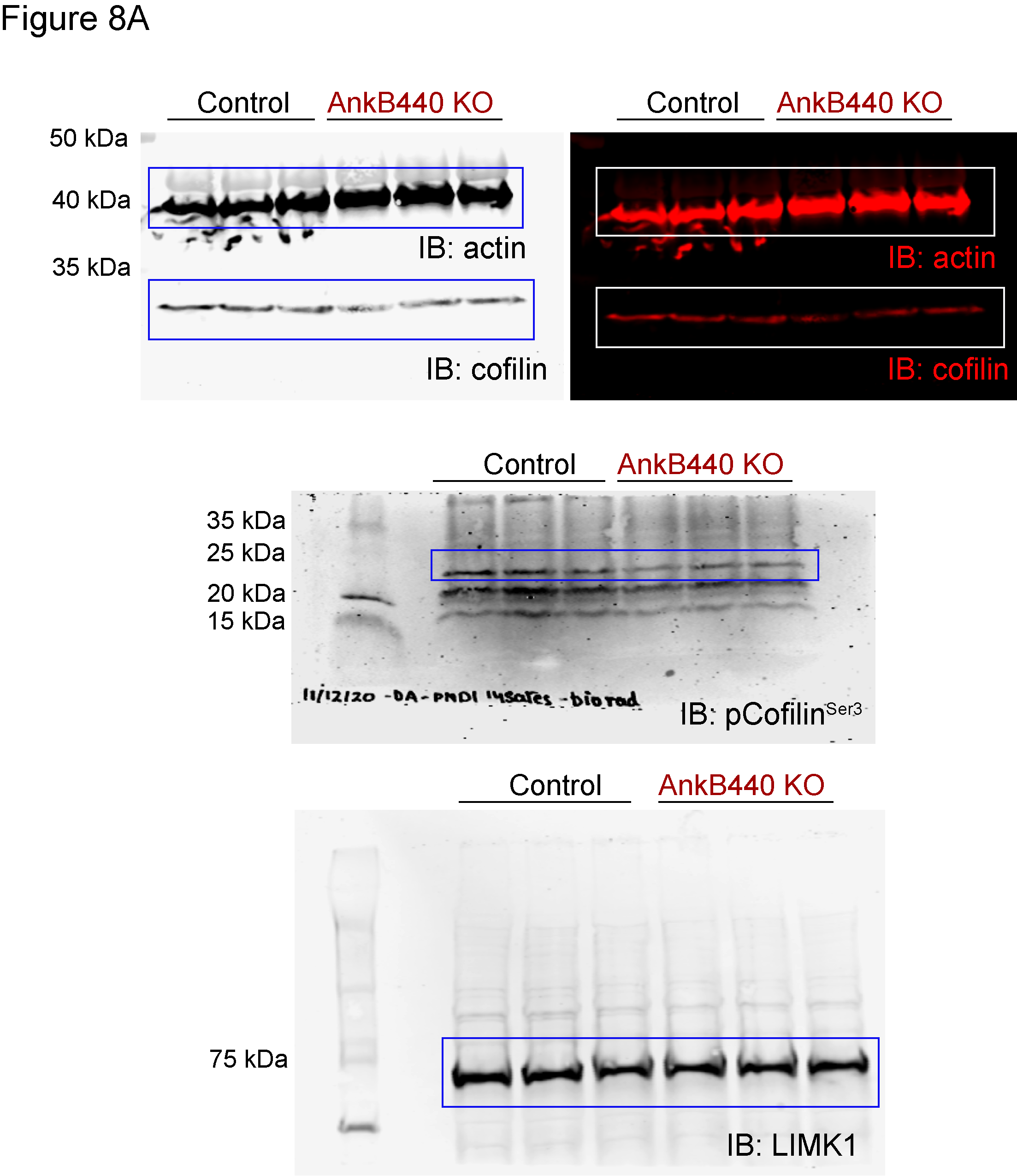

Supplement: Figure 8—source data 1. [file elife-69815-fig8-data1.zip › Figure 8-source data/Figure 8-souce data 5.tif]

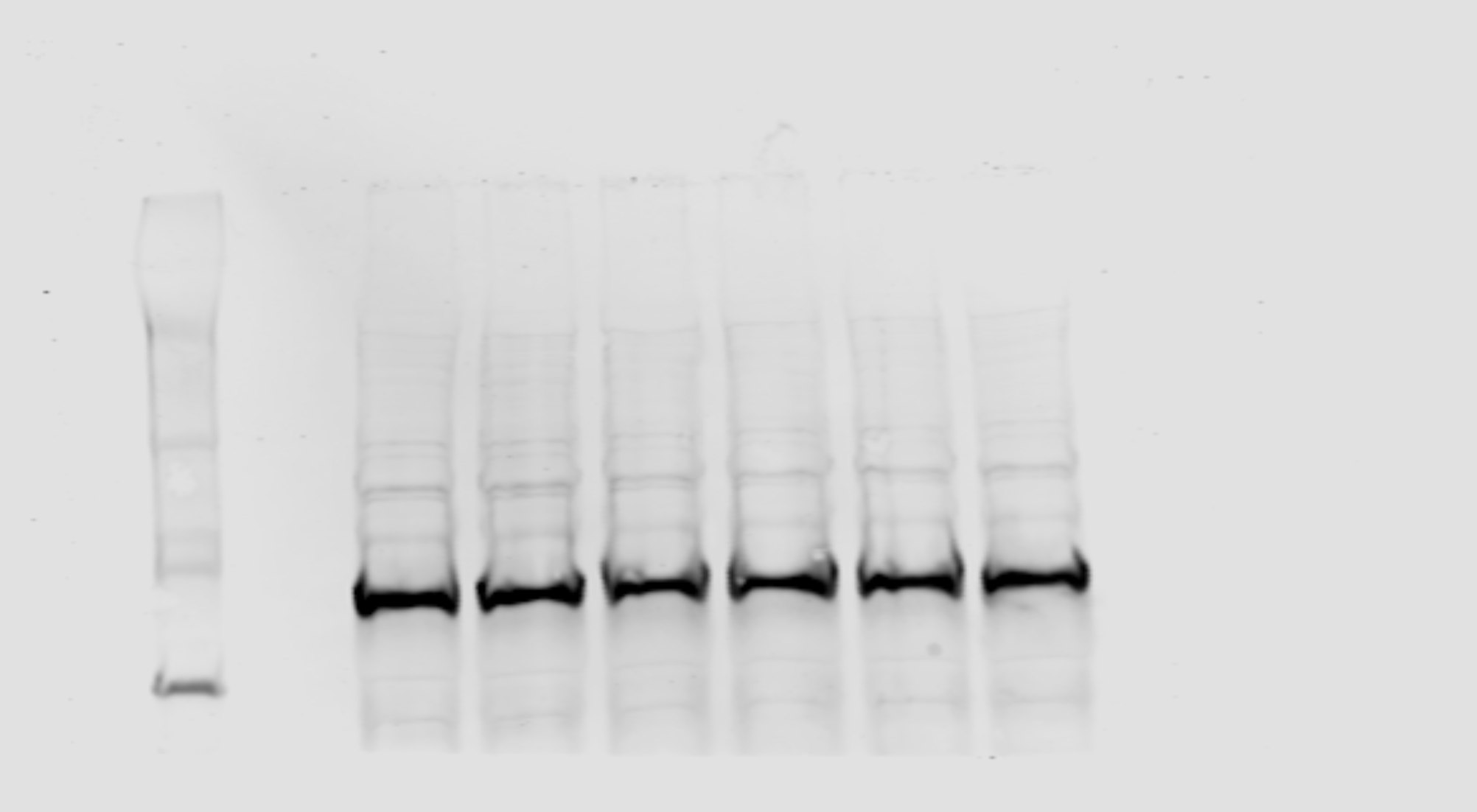

Supplement: Figure 8—source data 1. [file elife-69815-fig8-data1.zip › Figure 8-source data/Figure 8-source data 1.tif]

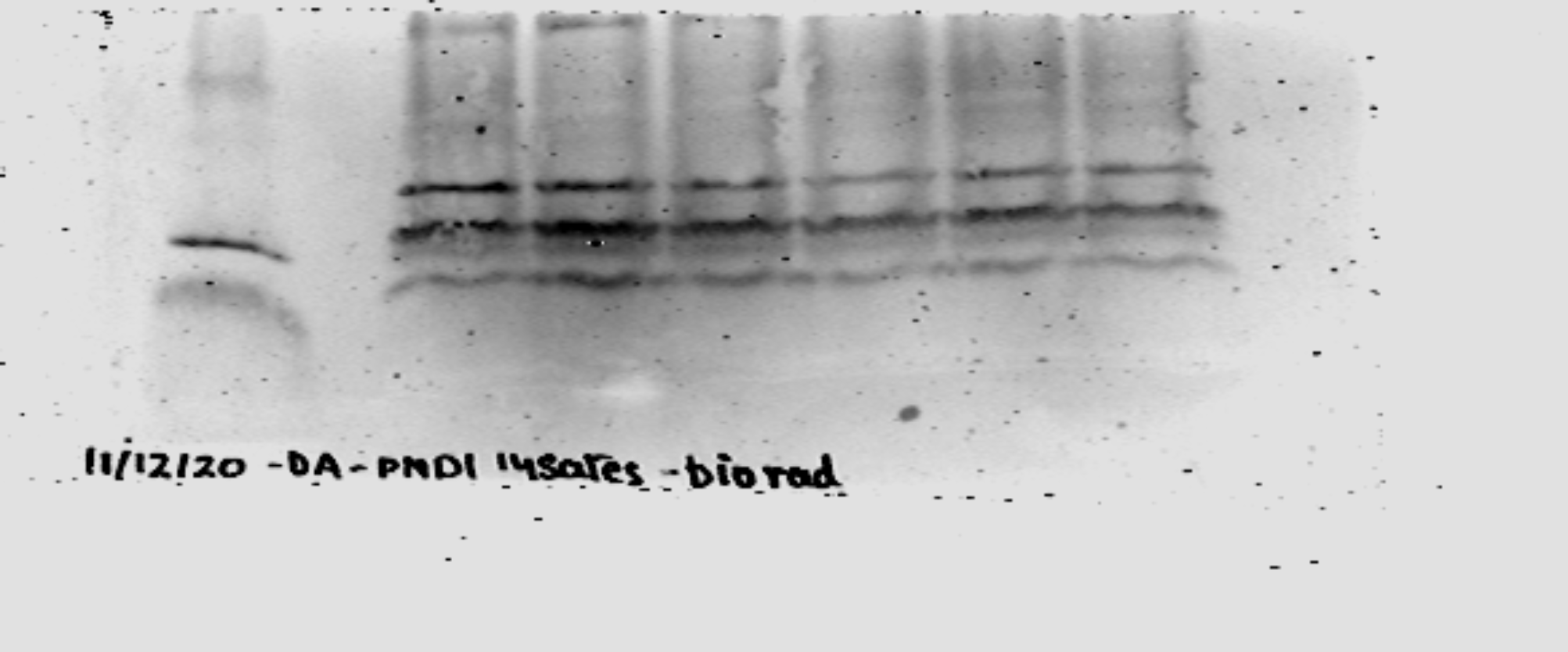

Supplement: Figure 8—source data 1. [file elife-69815-fig8-data1.zip › Figure 8-source data/Figure 8-source data 2.tif]

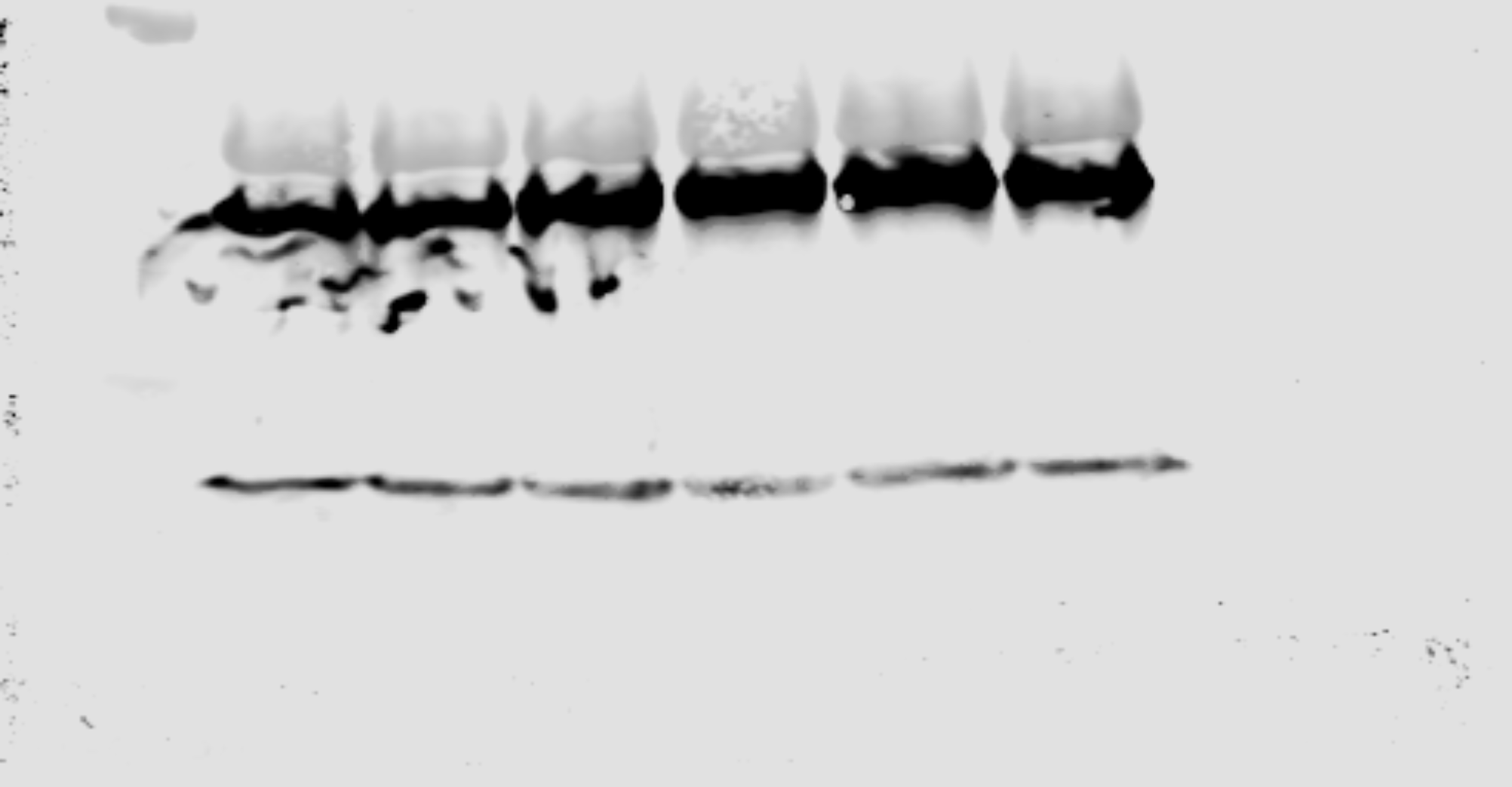

Supplement: Figure 8—source data 1. [file elife-69815-fig8-data1.zip › Figure 8-source data/Figure 8-source data 3.tif]

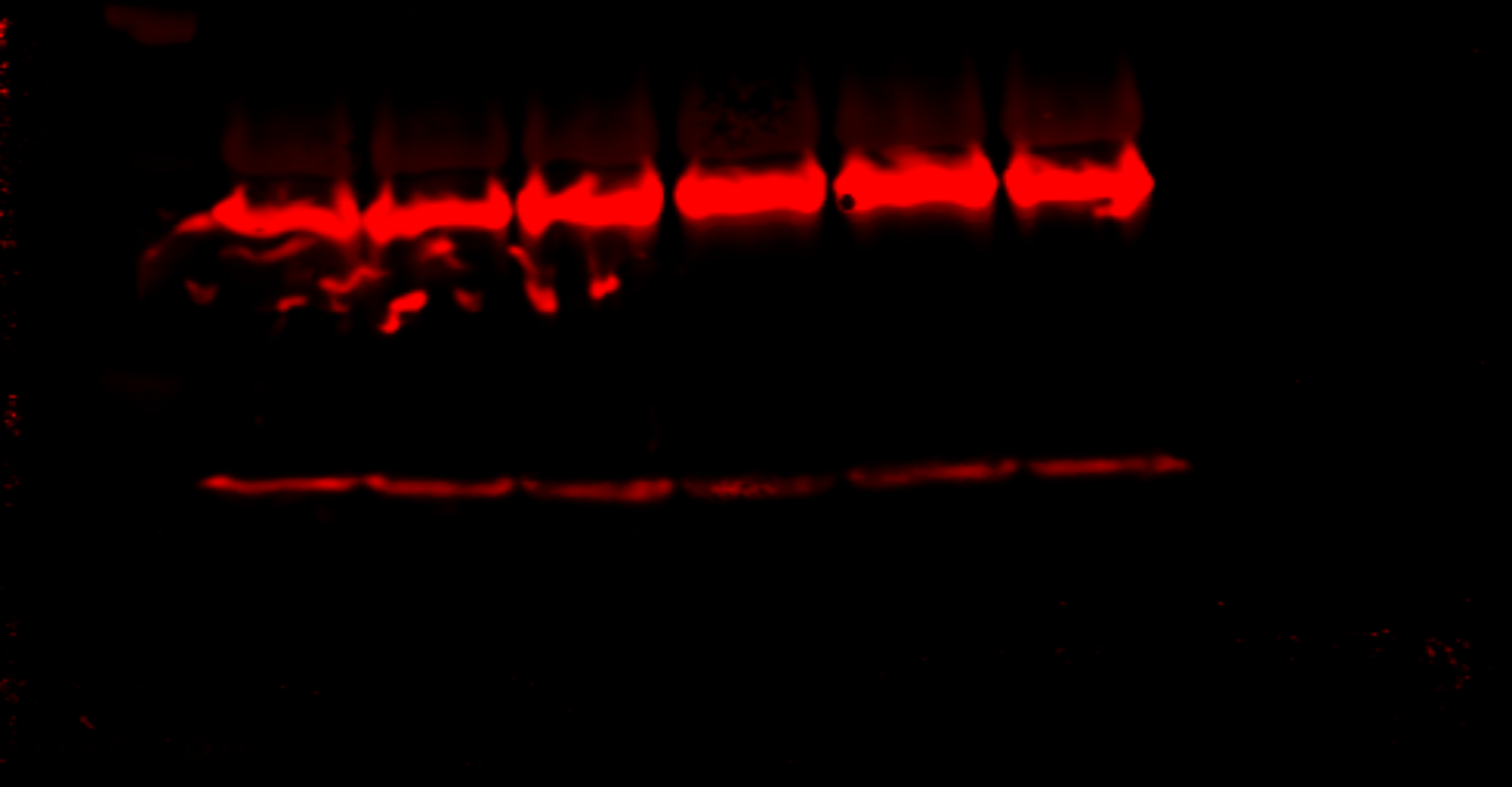

Supplement: Figure 8—source data 1. [file elife-69815-fig8-data1.zip › Figure 8-source data/Figure 8-source data 4.tif]

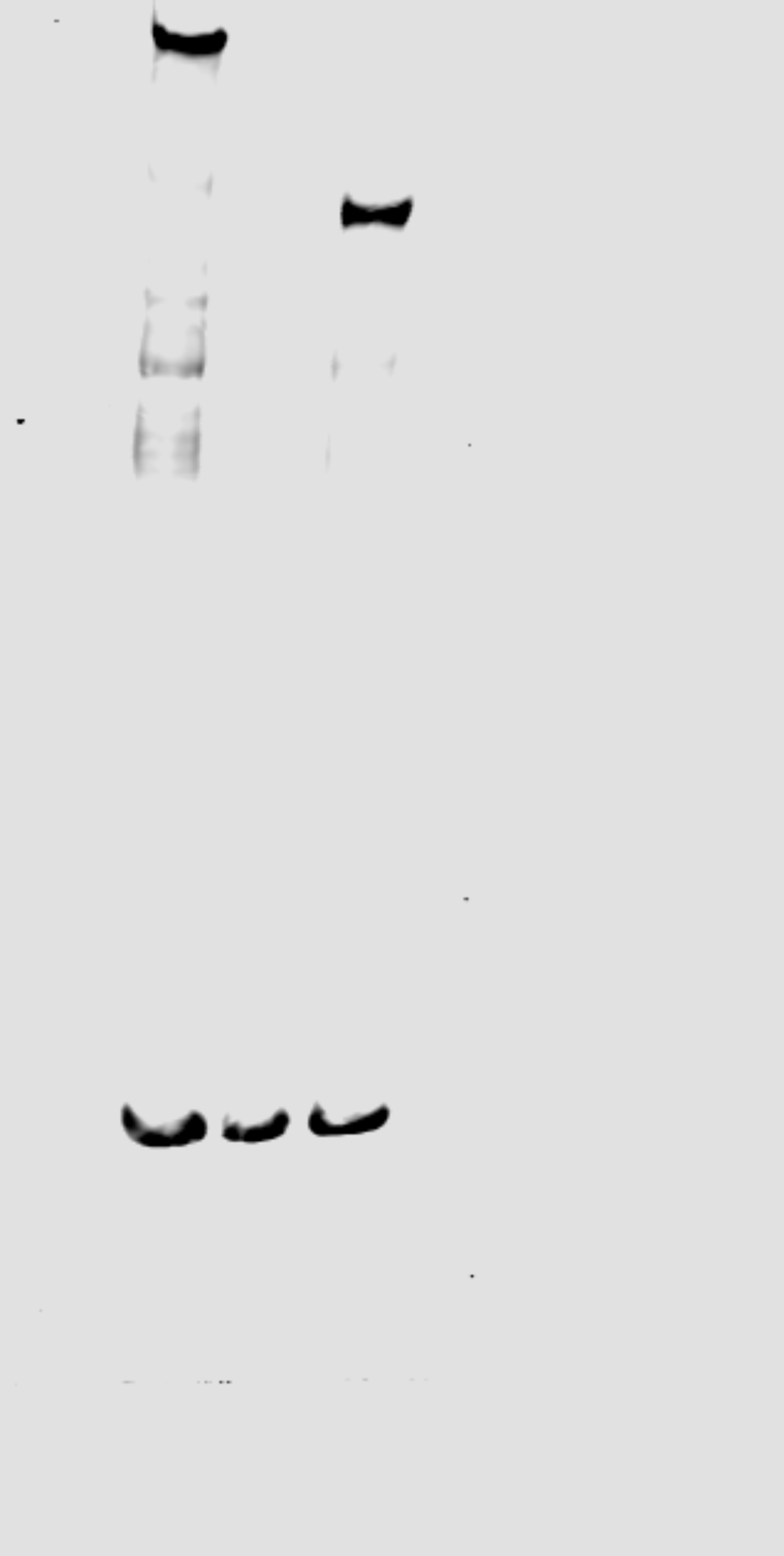

Supplement: Figure 9—source data 1. [file elife-69815-fig9-data1.zip › Figure 9-source data/Figure 9- source data 1.tif]

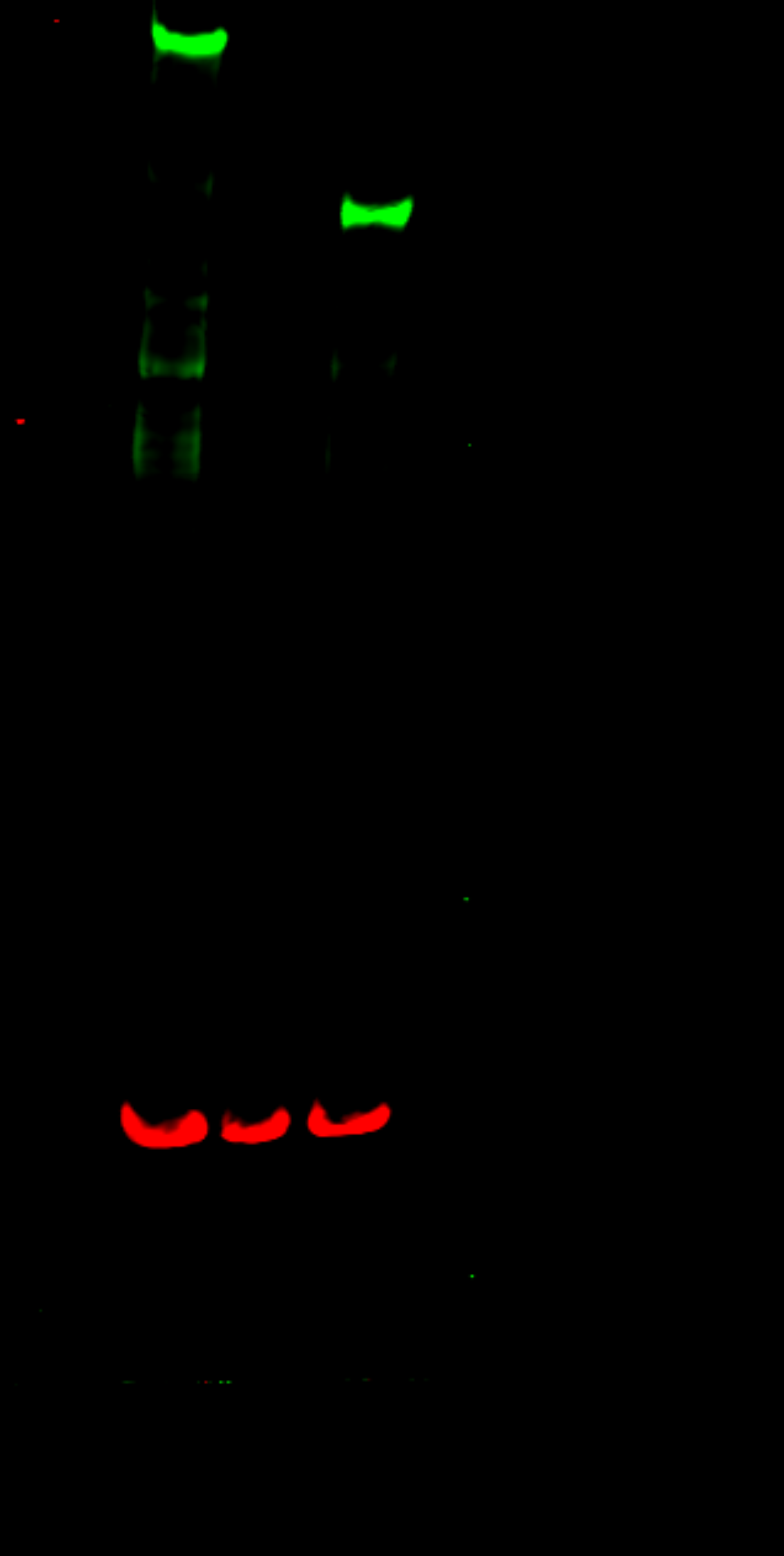

Supplement: Figure 9—source data 1. [file elife-69815-fig9-data1.zip › Figure 9-source data/Figure 9-source data 2.tif]

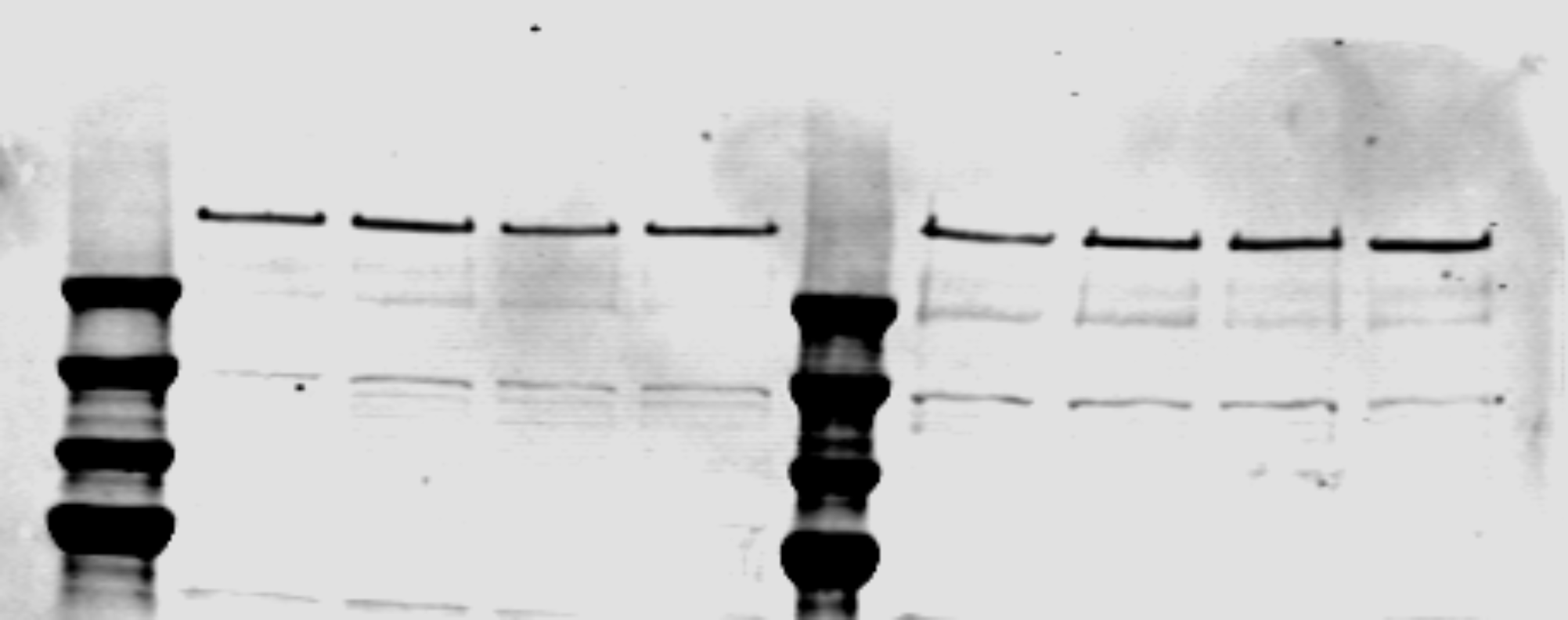

Supplement: Figure 9—source data 1. [file elife-69815-fig9-data1.zip › Figure 9-source data/Figure 9-source data 3.tif]

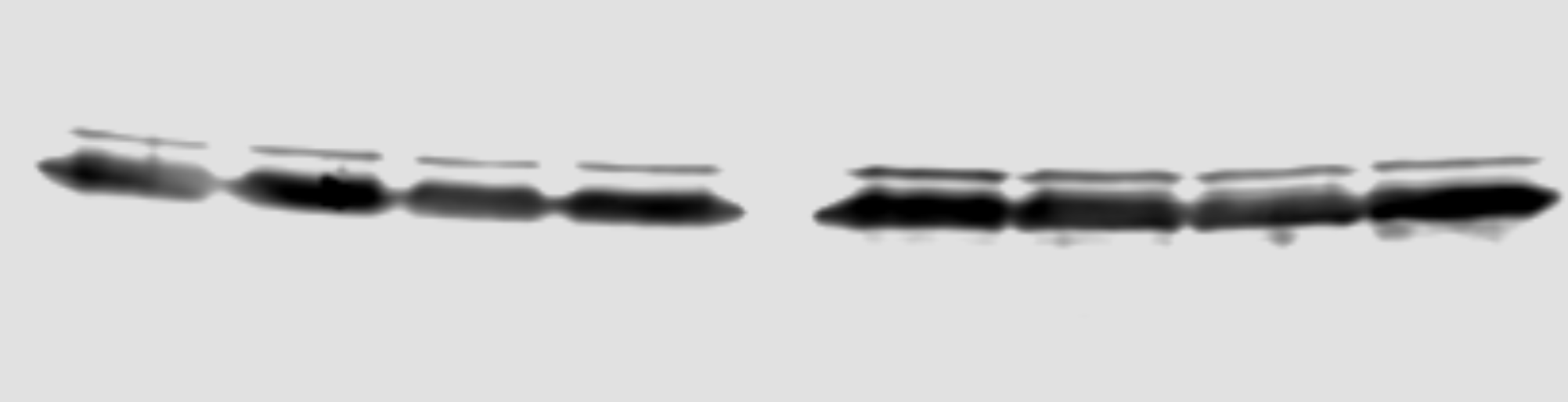

Supplement: Figure 9—source data 1. [file elife-69815-fig9-data1.zip › Figure 9-source data/Figure 9-source data 4.tif]

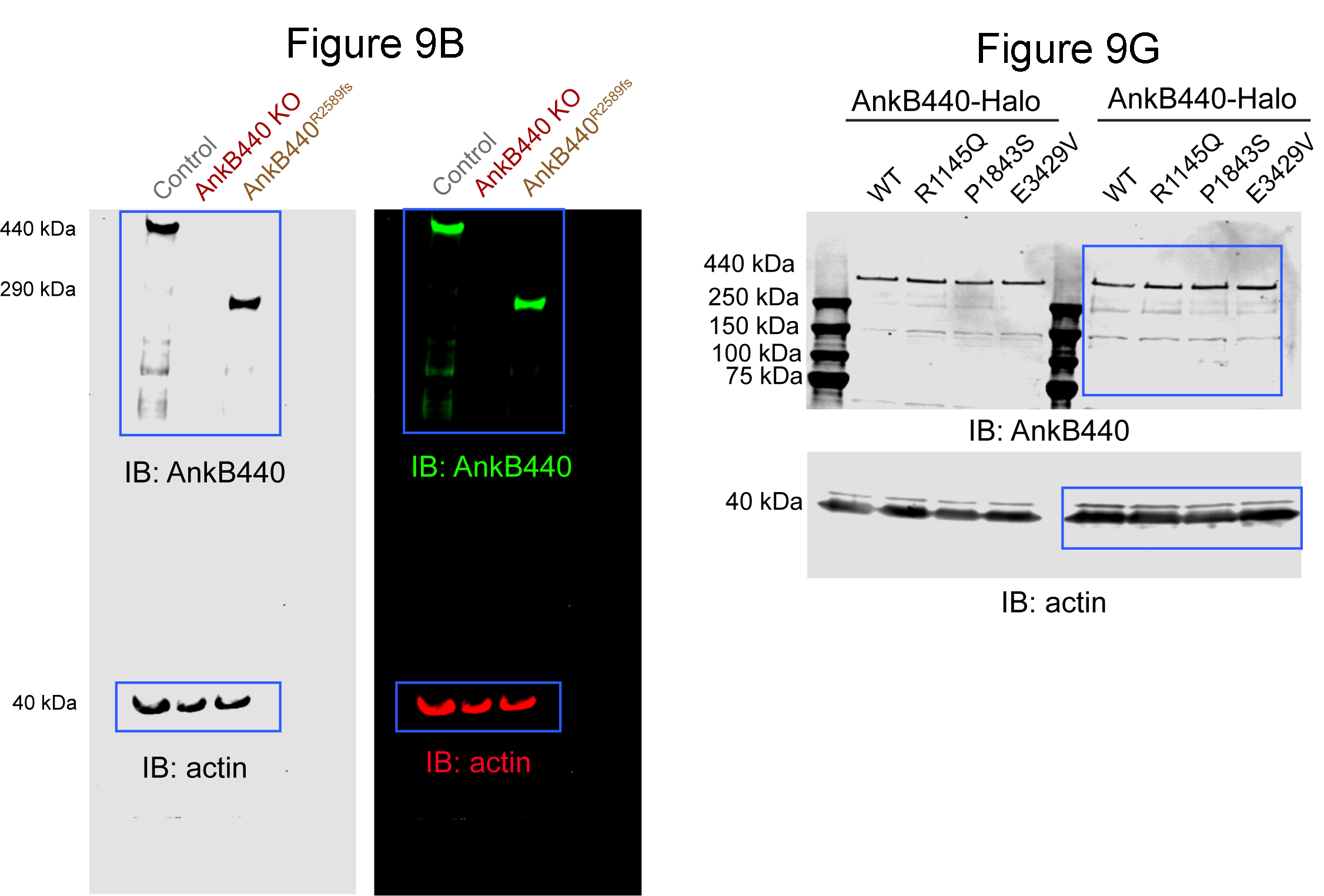

Supplement: Figure 9—source data 1. [file elife-69815-fig9-data1.zip › Figure 9-source data/Figure 9-source data 5.tif]
